# Supplementary material for: Quantitative, Wide-Spectrum Kinase Profiling in Live Cells for Assessing the Effect of Cellular ATP on Target Engagement
Source: Cell Chem Biol. 2018 Feb 15;25(2):206–214.e11. doi: 10.1016/j.chembiol.2017.10.010 (PMC5814754; doi:10.1016/j.chembiol.2017.10.010)
Supplement: Document S1. Figures S1–S7 and Table S2 [file mmc1.pdf]

Cell Chemical Biology, Volume *25*

Supplemental Information

Quantitative, Wide-Spectrum Kinase Profiling in Live Cells for Assessing the Effect of Cellular ATP on Target Engagement

James D. Vasta, Cesear R. Corona, Jennifer Wilkinson, Chad A. Zimprich, James R. Hartnett, Morgan R. Ingold, Kristopher Zimmerman, Thomas Machleidt, Thomas A. Kirkland, Kristin G. Huwiler, Rachel Friedman Ohana, Michael Slater, Paul Otto, Mei Cong, Carrow I. Wells, Benedict-Tilman Berger, Thomas Hanke, Carina Glas, Ke Ding, David H. Drewry, Kilian V.M. Huber, Timothy M. Willson, Stefan Knapp, Susanne Müller, Poncho L. Meisenheimer, Frank Fan, Keith V. Wood, and Matthew B. Robers

Supplemental Information

Quantitative, Wide-Spectrum Kinase Profiling in Live Cells for Assessing the Effect of Cellular ATP on Target Engagement

Content

Figure S1, Reproducibility of compound IC_{50} measurements for Kinase/NanoLuc fusions, Related to figure 4……2
Figure S2, Bioluminescence imaging of Nluc fusion proteins and CRISPR experiments, Related to figures 3–6……4
Figure S3, Analysis of chemical probe 6j, examination of the influence of tracer on test compound affinity for RIPK1, and reproducibility of crizotib profiling, Related to figures 3–6………………………6
Table S2, IC_{50} or K_d values for crizotinib under various assay conditions, Related to figure 5………………8
Figure S4, Reproducibility of kinome profiling with crizotinib and dasatinib, Related to figure 6……………9
Figure S5, Validation of ATP depletion conditions for determination of kinase target engagement, Related to figure 7……………………………………………………………………………………………………10
Figure S6, Determination of target engagement parameters for kinase/NLuc fusions under ATP depleted conditions, Related to figure 7………………………………………………………………………12
Figure S7, Determination of crizotinib or ATP affinity for LCK, EPHB6 and MuSK kinases in permeabilized HEK293 cells using linearized Cheng-Prusoff analysis, Related to figure 7…………14

Figure S1, Related to Figure 4

A

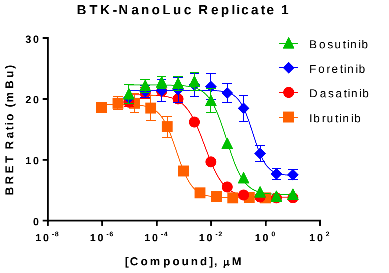

	Bosutinib	Foretinib	Dasatinib	Ibrutinib
LogEC50	0.04124	0.06707	0.02714	0.03892

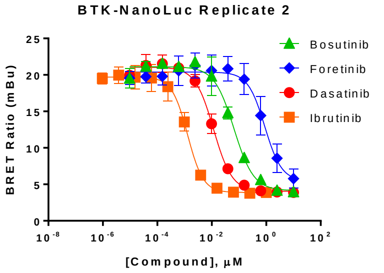

	Bosutinib	Foretinib	Dasatinib	Ibrutinib
EC50	0.06529	0.884	0.01164	0.001294

B

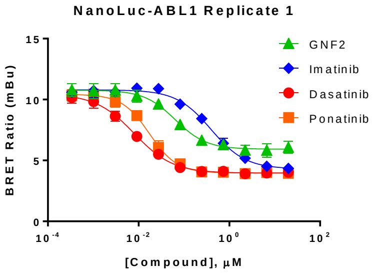

	GNF2	Imatinib	Dasatinib	Ponatinib
EC50	0.0642	0.3947	0.00803	0.01732

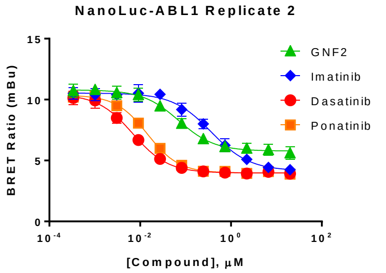

	GNF2	Imatinib	Dasatinib	Ponatinib
EC50	0.07024	0.3676	0.006847	0.01443

C

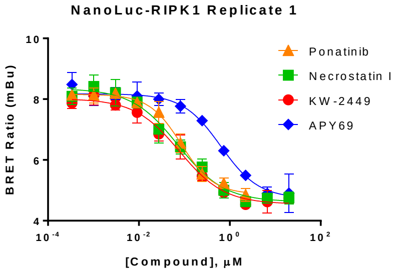

	APY69	KW-2449	Necrostatin I	Ponatinib
EC50	0.6339	0.07844	0.07311	0.08604

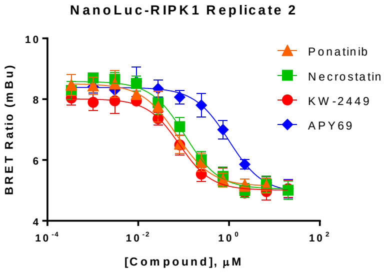

	APY69	KW-2449	Necrostatin I	Ponatinib
EC50	0.9828	0.08098	0.1069	0.07028

D

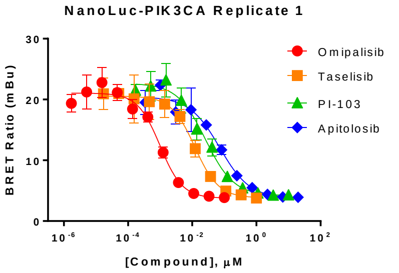

	Omipalisib	Taselisib	PI-103	Apitolosib
EC50	0.0009871	0.01203	0.02588	0.07154

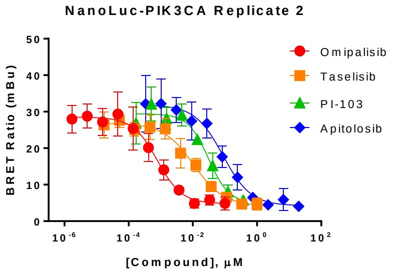

	Omipalisib	Taselisib	PI-103	Apitolosib
EC50	0.0007782	0.01057	0.03054	0.08633

E

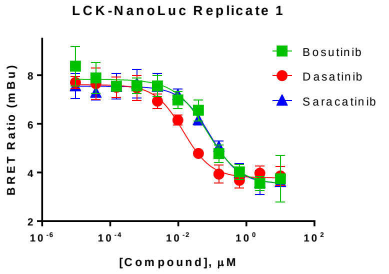

	Bosutinib	Dasatinib	Saracatinib
EC50	0.07079	0.01402	0.08623

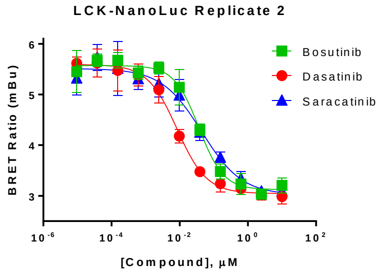

	Bosutinib	Dasatinib	Saracatinib
EC50	0.03662	0.008386	0.04445

Figure S1, Reproducibility of compound IC_{50} measurements for Kinase/NanoLuc Fusions, Related to Figure 4. HEK293 cells expressing the kinase/Nluc fusion were seeded into 96-well plates and then treated with a dose-response of compound in the presence of a fixed concentration of energy transfer probe (as described in supplemental table 1). Data in each curve represent the mean (± SD) of 4 technical replicates.

Figure S2, Related to Figures 3–6

A

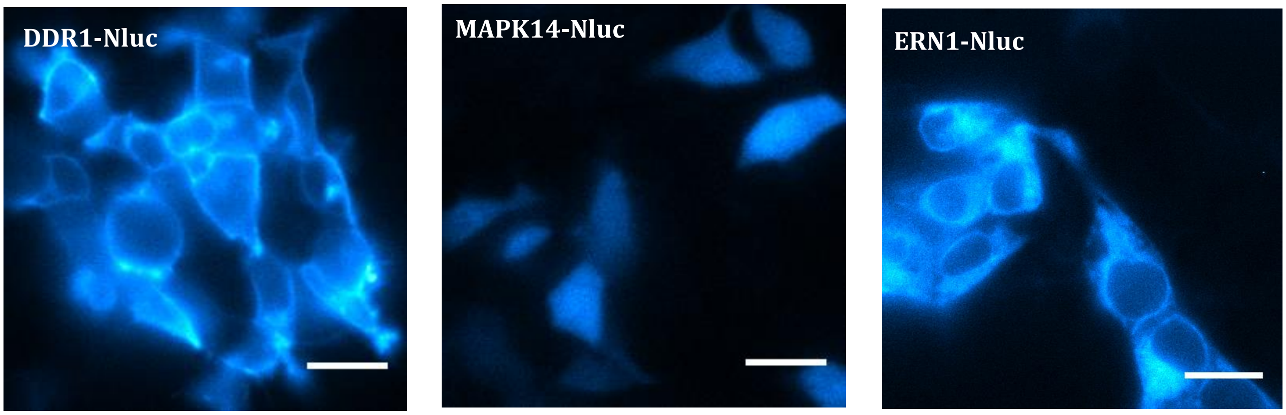

B

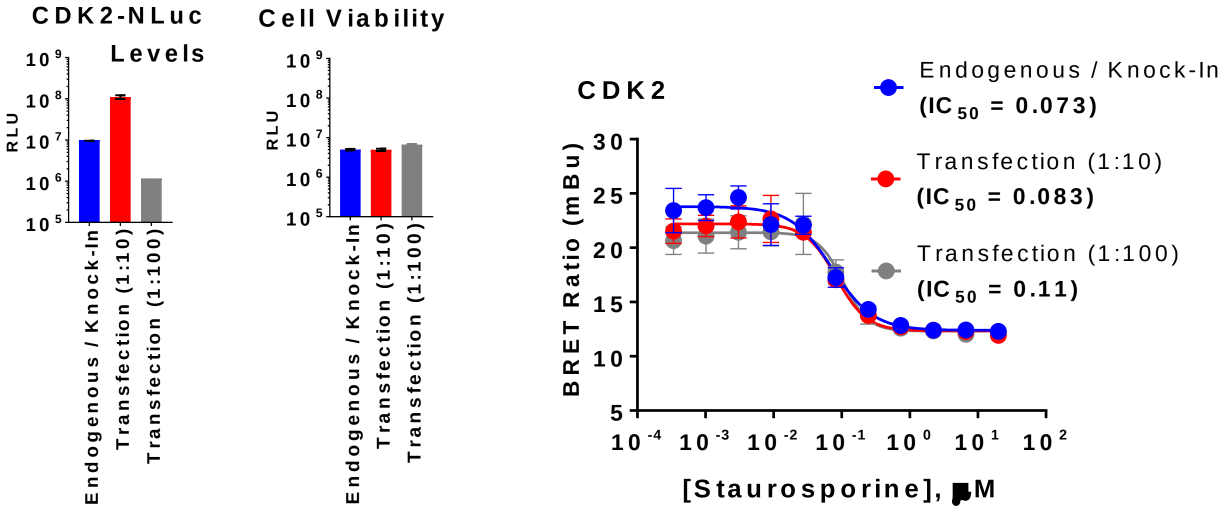

C

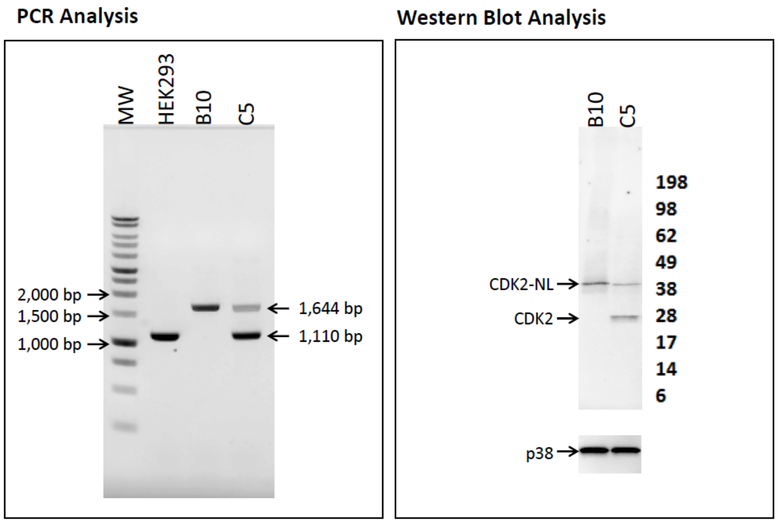

D

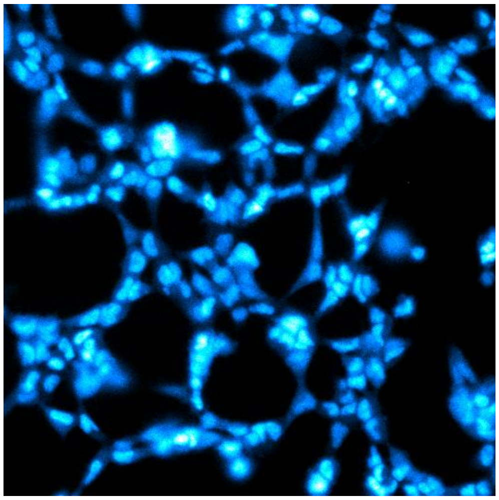

Figure S2. Bioluminescence imaging of Nluc fusion proteins and CRISPR experiments, Related to Figures 3–6. A. Bioluminescence imaging of Nluc fusion proteins. DDR1, MAPK14, and ERN1 (IRE1α) show localization in the plasma membrane, cytoplasm, or endoplasmic reticulum, respectively, thus supporting target engagement analysis in various subcellular compartments. Scale bar = 20 μm. B. Comparison of target engagement at CDK2 in HEK-293 cells, using endogenously expressed (CRISPR-edited) CDK2-NLuc, versus cells transiently expressing CDK2-Nluc proteins. Nluc expression levels with transfected cells were within 10-fold of the expression levels of endogenously-expressed (CRISPR-edited) CDK2, as measured using Nluc luminescence to quantify the relative amounts of CDK2-Nluc (left). To dilute expression levels of CDK2-Nluc, plasmid DNA was diluted into DNA encoding cyclin E from a pF5 plasmid backbone. Cell mass was similar under each condition, as determined using ATP levels via Cell-Titer-Glo (middle). Target engagement potencies with staurosporine were similar under all expression levels (right). Data are the mean (± SD) of 4 technical replicates. C: CRISPR-edited HEK-293 cells expressing CDK2-Nluc are homozygous (clone B10) based on PCR and Western blot analysis. The heterozygous clone (C5) is included for reference. D: In the CRISPR-derived CDK2-Nluc B10 clone, localization of Nluc was predominantly nuclear, consistent with expected localization of CDK2 (Hiromura et al., 2002). Localization was determined using bioluminescence imaging in live cells in the presence of NanoLuc substrate.

Figure S3, Related to Figures 3–6

A

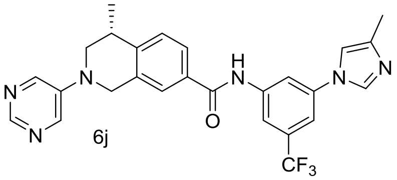

6j

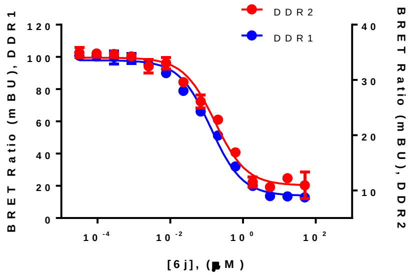

B

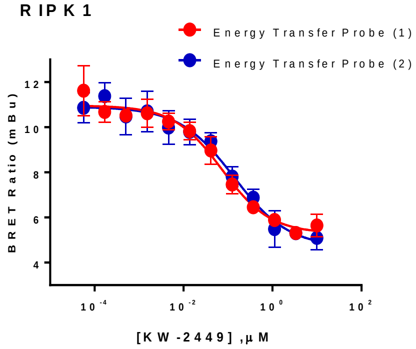

	Probe 1	Probe 2
IC50	0.07318	0.1361

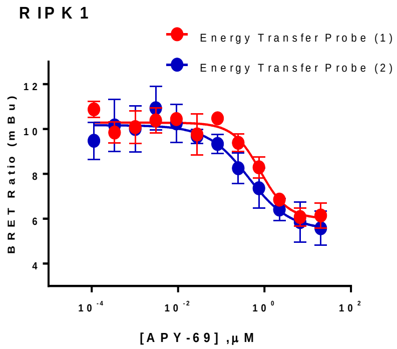

	Probe 1	Probe 2
IC50	0.7914	0.4283

C

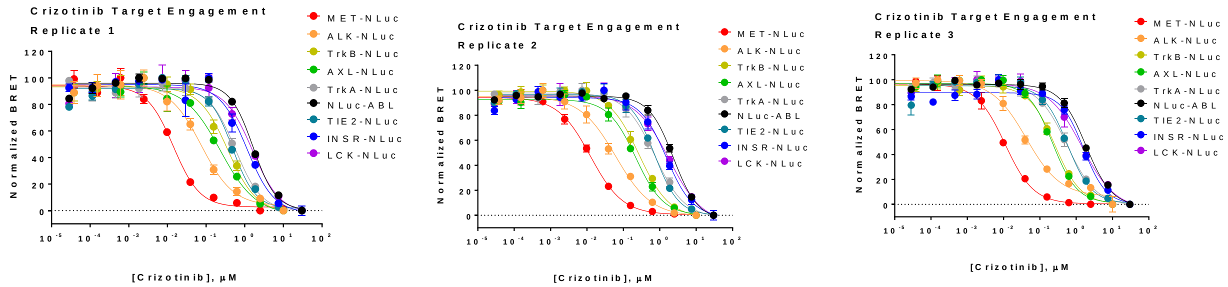

D

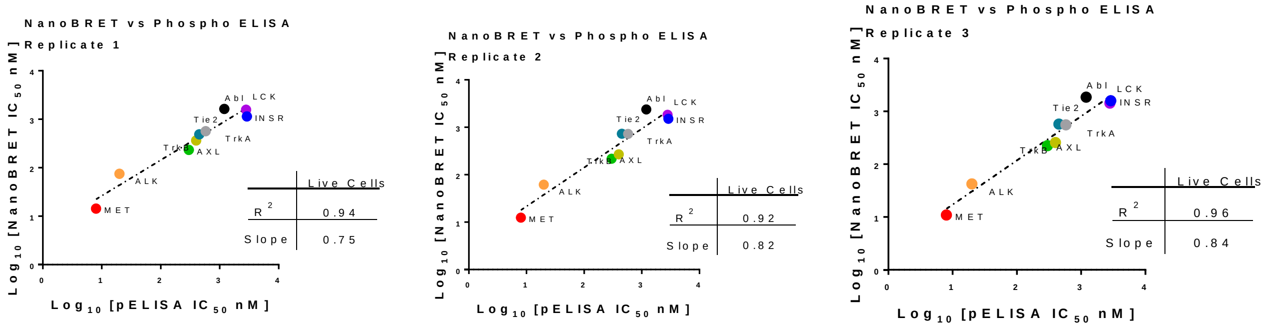

Figure S3. Analysis of chemical probe 6j, examination of the influence of tracer on test compound affinity for RIPK1, and reproducibility of crizotib profiling, Related to Figures 3–6. A. Chemical structure (left) and target engagement potency of 6j for DDR1 vs DDR2 (right) using energy transfer probe 6. Data are mean +/- S.E. for three independent experiments performed in 384-well format. B. Comparative target engagement analysis for RIPK1 using various energy transfer probes. For RIPK1, engagement potency was within a two-fold range for KW-2449 and APY-69 using energy transfer probe 1 or 2. Data are the mean (± SD) of 4 technical replicates. C. Reproducibility of crizotinib target engagement potency for various intracellular kinases. Full data set for crizotinib kinase panel represents three independent experiments, with IC_{50} values for crizotinib at each kinase target tabulated in Table S2. D. IC_{50} values extracted from curve fits are correlated with the corresponding crizotinib IC_{50} as determined by previously by Cui *et al.*, 2011, via phospho ELISA after log transformation.

Table S2, IC_{50} or K_d values for Crizotinib Under Various Assay Conditions, Related to Figure 5

Target	Crizotinib K_d Biochemical (nM) [A]	Crizotinib IC_{50} Biochemical (nM) [B]	Live Cell IC_{50} NanoBRET (nM)	Live Cell IC_{50} Phospho Elisa (nM) [B]
**ABL**	110	24	2000 ± 400	1200
**ALK**	3.3	< 1.0	60 ± 20	20
**AXL**	7.8	< 1.0	225 ± 8	300
**INSR**	340	102	1400 ± 200	2900
**TIE2**	270	5.0	600 ± 100	450
**TRKA**	95	< 1.0	600 ± 100	580
**TRKB**	37	2.0	300 ± 60	400
**LCK**	30	< 1.0	1600 ± 200	2800
**MET**	2.1	< 1.0	13 ± 2	8

[A]Data is reproduced from Davis *et al.,* 2011

[B]Data is reproduced from Cui *et al.,* 2011

Figure S4, Related to Figure 6

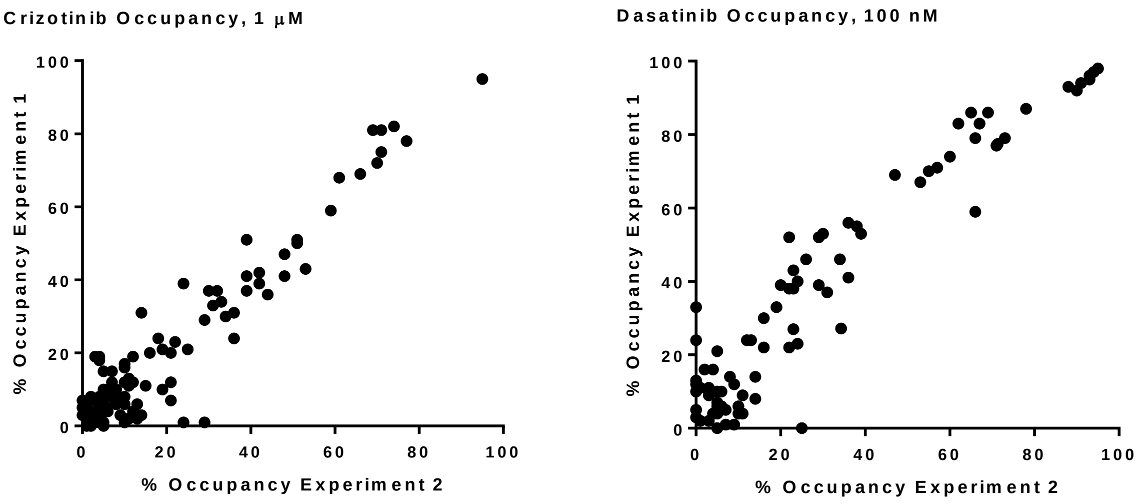

Figure S4, Reproducibility of kinome profiling with crizotinib and dasatinib, Related to Figure 6. HEK293 cells expressing a library of kinase/Nluc fusions were seeded into 96-well plates and then treated with 1 uM crizotinib (left) or 100 nM dasatinib (right) in the presence of energy transfer probe. Data in each graph represent the inter-assay correlation analysis of target occupancy for 2 independent experimental replicates.

Figure S5, Validation of ATP depletion conditions for determination of kinase target engagement, Related to Figure 7

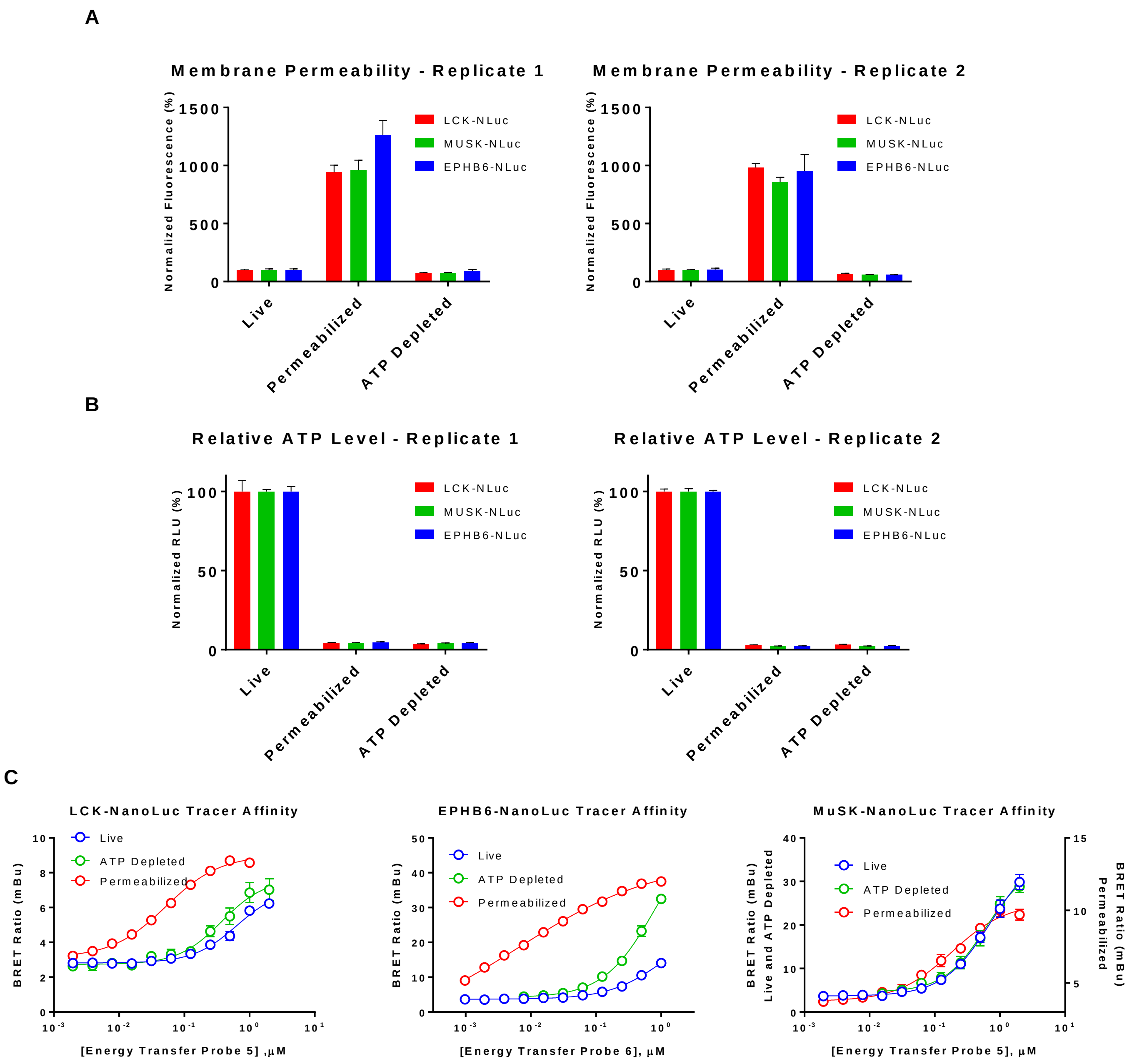

Condition	Live	ATP Depleted	Permeabilized
EC_{50}, μM	> 1	0.37 ± 0.07	0.052 ± 0.003

Condition	Live	ATP Depleted	Permeabilized
EC_{50}, μM	> 1	> 1	0.007 ± 0.003

Condition	Live	ATP Depleted	Permeabilized
EC_{50}, μM	> 1	> 1	0.18 ± 0.02

Figure S5, Validation of ATP depletion conditions for determination of kinase target engagement, Related to Figure 7. A. Validation of live-cell ATP depletion and cell permeabilization conditions**.** HEK293 cells transiently expression the kinase/NLuc fusion protein were subjected to ATP depletion by either inhibition of mitochondrial/glycolytic ATP synthesis (ATP Depleted) or by permeabilization with digitonin (Permeabilized) as described earlier. After treatment for 2h, membrane integrity (Panels A) and relative ATP levels (Panel B) were assessed using CellTox-Green and CellTiter-Glo assays, respectively. Raw fluorescence or luminescence results for all conditions were normalized to that of vehicle treated controls (Live). Individual data sets represent the mean (± SD) of 4 technical replicates. Replicates 1 and 2 represent the results of two independent experiments. Permeabilization with digitonin routinely disrupted membrane integrity (increased relative fluorescence) and reduced total ATP levels (reduced relative RLU) compared to untreated controls. Within 2 hours, ATP depletion by inhibition of mitochondrial/glycolytic ATP synthesis routinely reduced total ATP levels by 90-95% of that for the untreated controls, but without disrupting membrane integrity (no significant increase in relative fluorescence by CellTox-Green). Panel C. Target engagement measurements for crizotinib at kinase/NLuc fusions under ATP depleted conditions. HEK293 cells transiently expressing kinase/NLuc were subjected to vehicle treatment (live), ATP depletion, or permeabilization, after which the target engagement profile of crizotinib was evaluated. To determine a comparable test concentration for subsequent IC_{50} determination, the affinity of energy transfer probe 5 for LCK and MuSK, as well as the affinity of energy transfer probe 6 for EPHB6, was measured under all test conditions. Individual data points represent the mean (± SD) of 4 technical replicates.

Figure S6, Related to Figure 7

A

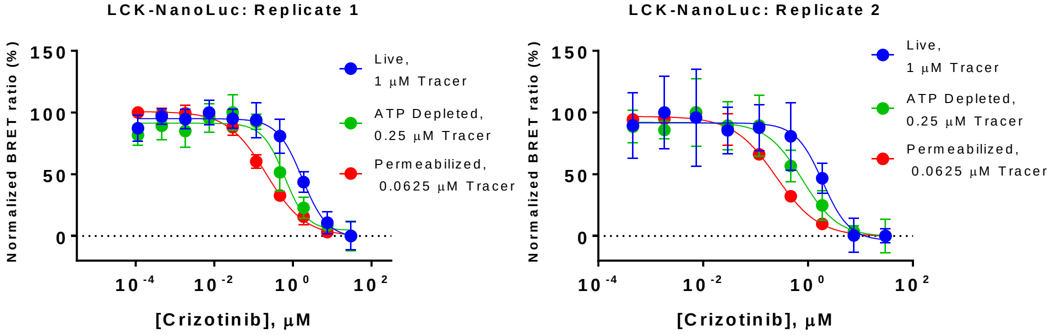

Condition	Live	ATP Depleted	Permeabilized
Crizotinib IC_{50}, nM	1700 ± 300	610 ± 140	210 ± 20

Condition	Live	ATP Depleted	Permeabilized
Crizotinib IC_{50}, nM	1900 ± 600	780 ± 270	250 ± 20

B

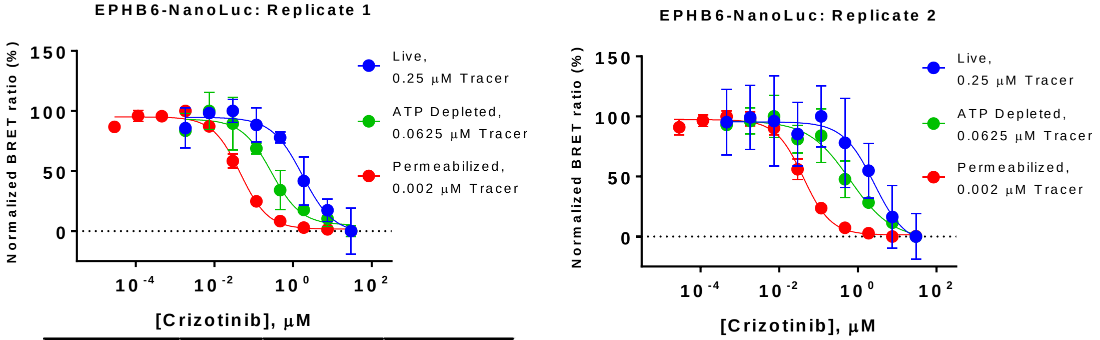

Condition	Live	ATP Depleted	Permeabilized
Crizotinib IC_{50}, nM	1700 ± 600	280 ± 90	45 ± 4

Condition	Live	ATP Depleted	Permeabilized
Crizotinib IC_{50}, nM	3000 ± 2000	630 ± 260	41 ± 4

C

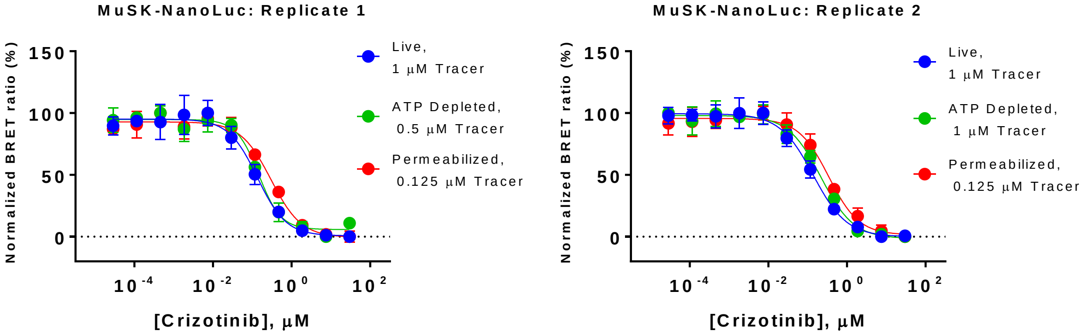

Condition	Live	ATP Depleted	Permeabilized
Crizotinib IC_{50}, nM	140 ± 20	150 ± 20	300 ± 40

Condition	Live	ATP Depleted	Permeabilized
Crizotinib IC_{50}, nM	140 ± 20	220 ± 30	300 ± 40

D

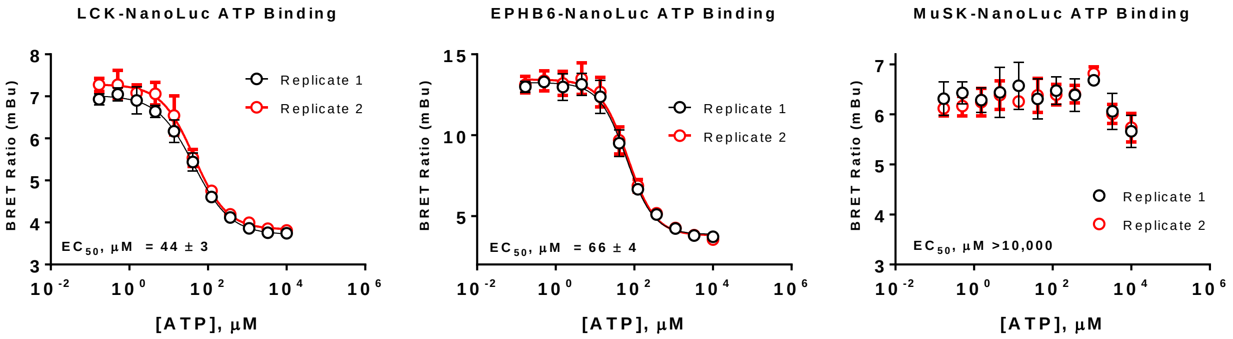

Figure S6. Determination of target engagement parameters for kinase/NLuc fusions under ATP depleted conditions, Related to Figure 7. Panels A–C. Crizotinib IC_{50} measurements for LCK (A) and EPHB6 (B) under ATP depleted and permeabilized conditions showed significant left shifted potency compared to the live cell controls, but that of MuSK (C) was consistent between 3 conditions. Individual data points represent the mean (± SD) of 4 technical replicates, with replicates 1 and 2 depicting the results of two independent measurements. D. LCK (Panel and EPHB6 showed significant ATP binding in permeabilized cells, but MuSK did not. Individual data points represent the mean (± SD) of 4 technical replicates. Replicates 1 and 2 represent the results of two independent experiments.

Figure S7, Related to Figure 7

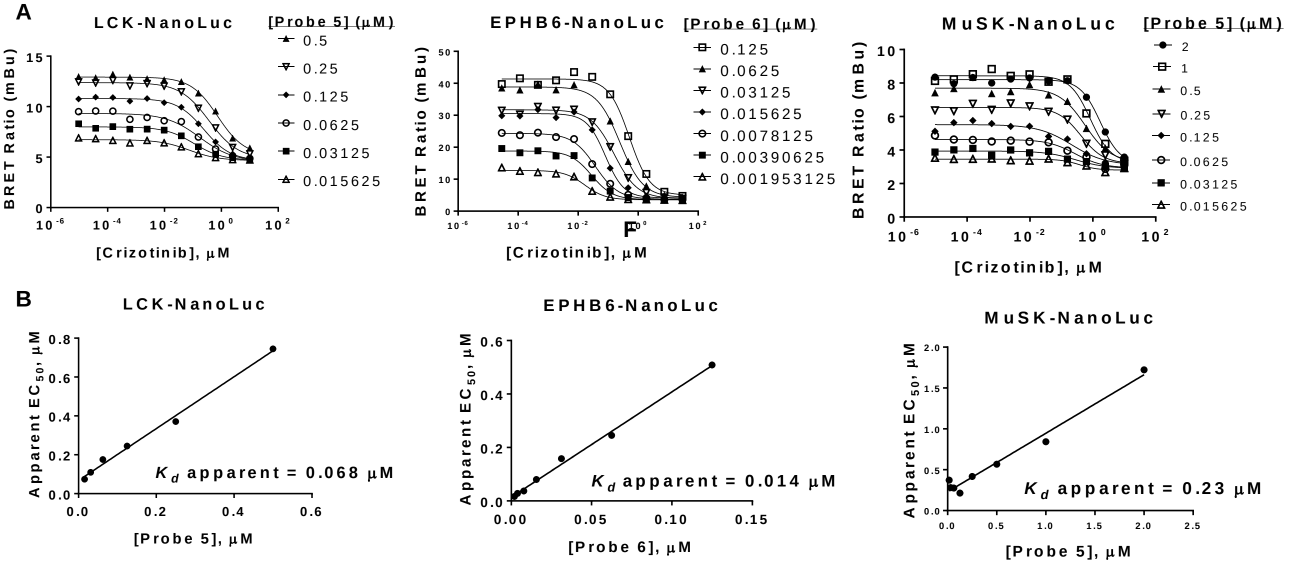

Target	LCK	EPHB6	MuSK
Crizotinib K_d, nM<br>Permeabilized HEK293 Cells	70 ± 10	14 ± 6	230 ± 40
Crizotinib K_i, nM<br>Biochemical (Davis, *et al*. 2011)	30	6	230

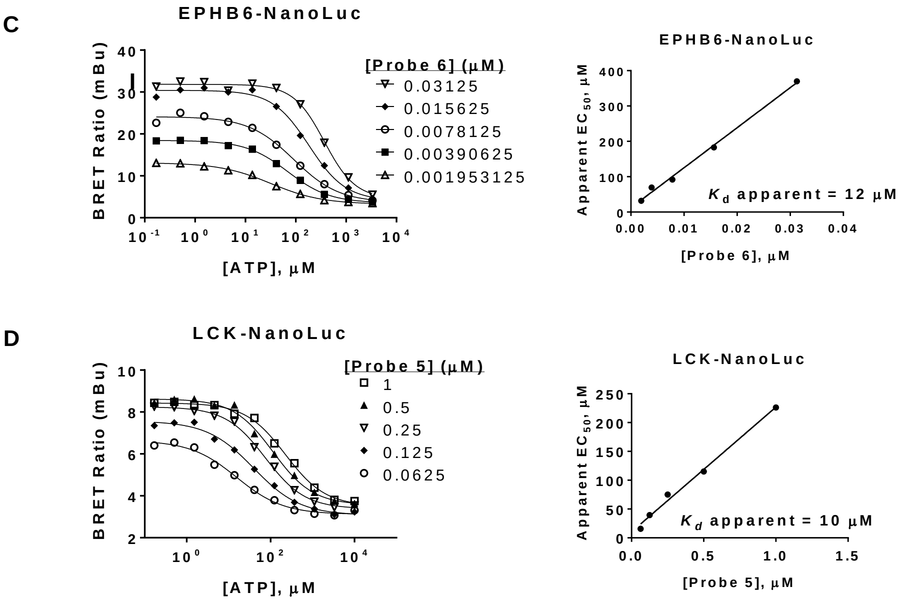

LCK Replicate	1	2	3	4	Mean
ATP K_d (μM)	10.49	19.62	15.37	15.69	15
Error	5.51	5.06	7.54	5.96	3

Figure S7, Determination of crizotinib or ATP affinity for LCK, EPHB6 and MuSK kinases in permeabilized HEK293 cells using linearized Cheng-Prusoff analysis, Related to Figure 7. A. Determination of crizotinib affinity. HEK293 cells expressing kinase/NanoLuc fusions were seeded into 96-well plates, permeabilized with digitonin, and then treated with a dose-response of crizotinib at increasing concentrations of energy transfer probe. (Panel B) Apparent IC_{50} values for crizotinib were replotted versus the energy transfer probe concentration, and the K_d value for crizotinib was determined from the y-intercept by linear regression. Individual data points are the mean (± SD) of 4 technical replicates. K_d values (± SE) for crizotinib are provided in the accompanying table, and are in agreement with biochemical K_i values reported previously (Davis *et al*., 2011). (Panel C) Determination of ATP affinity for EPHB6-NanoLuc. HEK293 cells expressing EPHB6-NanoLuc were seeded into 96-well plates, permeabilized with digitonin, and then treated with a dose-response of rATP at increasing concentrations of energy transfer probe 6. Apparent IC_{50} values for rATP were replotted versus the energy transfer probe concentration, and the K_d value for rATP was determined from the y-intercept by linear regression. Though a pseudokinase, EPHB6 has been shown previously to bind rATP using a thermal shift assay (Murphy *et al*., 2014). (Panel D) Determination of ATP affinity for LCK-NanoLuc. HEK293 cells expressing LCK-NanoLuc were seeded into 96-well plates, permeabilized with digitonin, and then treated with a dose-response of rATP at increasing concentrations of energy transfer probe 5 (1–0.0625 μM). Apparent IC_{50} values for rATP were replotted versus the energy transfer probe concentration, and the K_d value for rATP was determined from the y-intercept by linear regression. Replicates 1–4 represent 4 independent experiments, and the individual K_d values for rATP are provided in the accompanying table. The mean K_d of ATP for LCK-NanoLuc is in agreement with the biochemical ATP K_M reported previously (Knight and Shokat, 2005).
