# Supplementary material for: Quantitative, Wide-Spectrum Kinase Profiling in Live Cells for Assessing the Effect of Cellular ATP on Target Engagement
Source: Cell Chem Biol. 2018 Feb 15;25(2):206–214.e11. doi: 10.1016/j.chembiol.2017.10.010 (PMC5814754; doi:10.1016/j.chembiol.2017.10.010)
Supplement: Document S2. Article plus Supplemental Information [file mmc3.pdf]

Resource

Cell Chemical Biology

Quantitative, Wide-Spectrum Kinase Profiling in Live Cells for Assessing the Effect of Cellular ATP on Target Engagement

Graphical Abstract

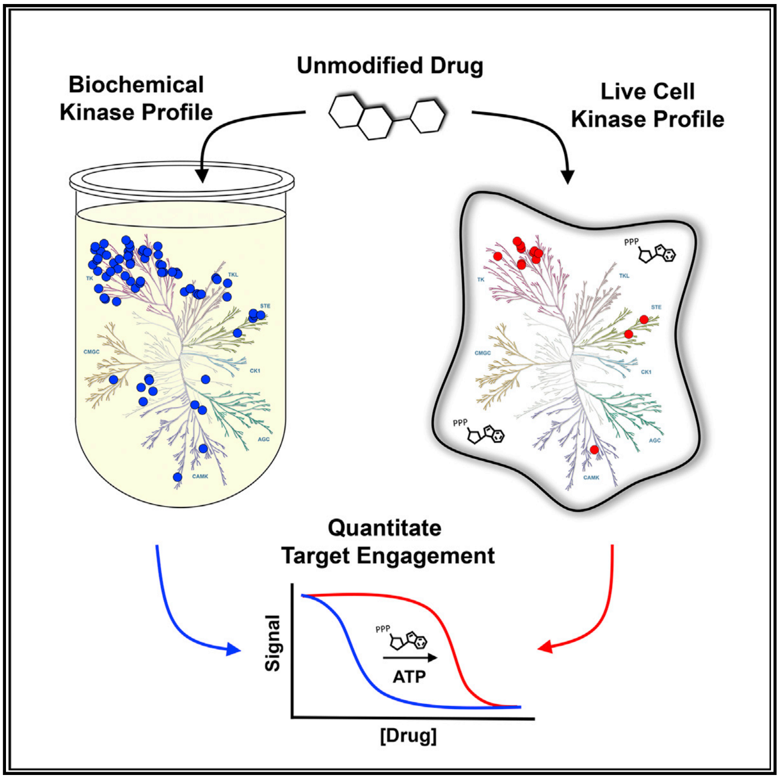

Highlights

- The approach enables quantitative profiling of 178 full-length kinases
- Compared with biochemical approaches, this is a better predictor of cellular potency
- An unexpected intracellular selectivity is observed for certain kinase inhibitors
- A mechanistic analysis of ATP interference on target engagement is performed

Authors

James D. Vasta, Cesear R. Corona, Jennifer Wilkinson, ..., Frank Fan, Keith V. Wood, Matthew B. Robers

Correspondence

matt.robers@promega.com

In Brief

Vasta et al. describe a broad-spectrum approach (BRET) to quantitatively measure target engagement for kinases inside live cells. Compared with biochemical measurements, the analysis revealed an improved intracellular selectivity profile for clinically relevant kinase inhibitors. This serves as a mechanistic tool to determine the effect of cellular ATP on engagement potency.

Vasta et al., 2018, Cell Chemical Biology *25*, 206–214
February 15, 2018 © 2017 The Author(s). Published by Elsevier Ltd.
https://doi.org/10.1016/j.chembiol.2017.10.010

CellPress

Cell Chemical Biology

Resource

Quantitative, Wide-Spectrum Kinase Profiling in Live Cells for Assessing the Effect of Cellular ATP on Target Engagement

James D. Vasta,[1] Cesear R. Corona,[2] Jennifer Wilkinson,[1] Chad A. Zimprich,[1] James R. Hartnett,[1] Morgan R. Ingold,[1] Kristopher Zimmerman,[1] Thomas Machleidt,[1] Thomas A. Kirkland,[2] Kristin G. Huwiler,[1] Rachel Friedman Ohana,[1] Michael Slater,[1] Paul Otto,[1] Mei Cong,[1] Carrow I. Wells,[3] Benedict-Tilman Berger,[4,5] Thomas Hanke,[4] Carina Glas,[6,7] Ke Ding,[8,9] David H. Drewry,[3] Kilian V.M. Huber,[6,7] Timothy M. Willson,[3] Stefan Knapp,[4,5] Susanne Müller,[5] Poncho L. Meisenheimer,[2] Frank Fan,[1] Keith V. Wood,[1] and Matthew B. Robers[1,10,*]

[1]Promega Corporation, 2800 Woods Hollow Road, Fitchburg, WI 53711, USA
[2]Promega Biosciences Incorporated, 277 Granada Drive, San Luis Obispo, CA 93401, USA
[3]Structural Genomics Consortium, UNC Eshelman School of Pharmacy, University of North Carolina at Chapel Hill, Chapel Hill, NC, USA
[4]Structural Genomics Consortium, Institute for Pharmaceutical Chemistry, Johann Wolfgang Goethe-University, Max-von-Laue-Straße 9, 60438 Frankfurt am Main, Germany
[5]Structural Genomics Consortium, Buchmann Institute for Molecular Life Sciences, Johann Wolfgang Goethe-University, Max-von-Laue-Straße 15, 60438 Frankfurt am Main, Germany
[6]Structural Genomics Consortium, Nuffield Department of Medicine, University of Oxford, Oxford, UK
[7]Target Discovery Institute, Nuffield Department of Medicine, University of Oxford, Oxford, UK
[8]State Key Laboratory of Respiratory Diseases, Guangzhou Institutes of Biomedicine and Health, Chinese Academy of Sciences, 190 Kaiyuan Avenue, Guangzhou 510530, China
[9]School of Pharmacy, Jinan University, 601 Huangpu Avenue West, Guangzhou 510632, China
[10]Lead Contact
*Correspondence: matt.robers@promega.com
https://doi.org/10.1016/j.chembiol.2017.10.010

SUMMARY

For kinase inhibitors, intracellular target selectivity is fundamental to pharmacological mechanism. Although a number of acellular techniques have been developed to measure kinase binding or enzymatic inhibition, such approaches can fail to accurately predict engagement in cells. Here we report the application of an energy transfer technique that enabled the first broad-spectrum, equilibrium-based approach to quantitatively profile target occupancy and compound affinity in live cells. Using this method, we performed a selectivity profiling for clinically relevant kinase inhibitors against 178 full-length kinases, and a mechanistic interrogation of the potency offsets observed between cellular and biochemical analysis. For the multikinase inhibitor crizotinib, our approach accurately predicted cellular potency and revealed improved target selectivity compared with biochemical measurements. Due to cellular ATP, a number of putative crizotinib targets are unexpectedly disengaged in live cells at a clinically relevant drug dose.

INTRODUCTION

Kinases represent the largest group of enzymes in the human proteome and are functionally integral to signal transduction. Dysregulated kinase activity is commonly associated with a number of human cancers and autoimmune diseases (Wu et al., 2016). Consequently, the human kinome represents one of the broadest sources of therapeutic targets for small-molecule modulation.

The majority of clinically relevant kinase inhibitors engage their target proteins at the kinase active site, and compete with high intracellular concentrations (1–10 mM) of the cosubstrate ATP (Wu et al., 2016; Becher et al., 2013). Selective modulation of individual kinases represents a challenge due to the highly conserved structure of the ATP binding pocket, most notably within the protein kinase family. Engagement of off-target kinases is therefore commonly observed, and collateral engagement is often implicated in drug side effects (Arrowsmith et al., 2015). Kinase inhibitors may also demonstrate polypharmacology over a number of intracellular targets, a feature that often underlies the therapeutic potential of lead drug candidates. As our understanding of the therapeutic relevance of the human kinome deepens, a growing need has emerged for methods capable of quantifying kinase inhibitor occupancy, selectivity, and affinity within the cellular environment where engagement would naturally occur.

Traditional biochemical techniques may fail to predict kinase engagement in cells (Smyth and Collins, 2009). Although methods with isolated proteins are generally robust and quantitative, they do not measure compound permeability, nor encompass the physiological complexity of the full-length kinase protein in the presence of the cellular milieu and regulatory circuits that modulate kinase function (Smyth and Collins, 2009). For example, reliance on isolated catalytic kinase domains may pose certain challenges for assessing the binding characteristics of allosteric inhibitors that occupy domains distal to the ATP pocket (i.e., type III/IV inhibitors) or for inhibitors that

 © 2017 The Author(s). Published by Elsevier Ltd.
This is an open access article under the CC BY license (http://creativecommons.org/licenses/by/4.0/).

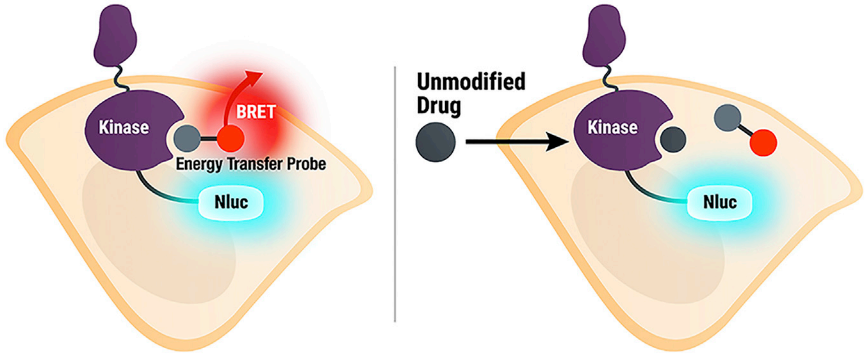

Figure 1. Illustration of Quantitative Energy Transfer Assay in Live Cells

Cell-permeable energy transfer probes reversibly engage the kinase active site, generating a BRET signal in live cells. Upon binding of an unmodified test compound to the selected kinase, the BRET signal is attenuated.

show activation-state-dependent engagement (Smyth and Collins, 2009; Wodicka et al., 2010; Cui et al., 2011; Garuti et al., 2010). Moreover, acellular approaches operate under defined assay conditions, typically at subsaturating concentrations of ATP. Intracellular ATP concentrations are likely non-uniform (exceeding 1 mM in some cellular compartments), and the affinity (i.e., K_M) of most kinases for ATP resides in the 10–100 μM range (Becher et al., 2013; Niki et al., 1989; Imamura et al., 2009). The interdependence of these factors leads to a challenge for use of biochemical assays as accurate proxies for cellular kinase engagement studies. Consequently, engagement potencies for ATP-competitive kinase inhibitors are often markedly shifted for their cellular targets, and biochemical kinase methodologies may fail to accurately predict cellular potency (Knight and Shokat, 2005; Smyth and Collins, 2009). A number of newer approaches have therefore been developed in an attempt to more accurately assess compound engagement at protein kinases expressed in human cells.

Mass spectrometry (MS)-based chemoproteomics approaches that utilize kinase inhibitor matrices may offer a more physiologically relevant assessment of kinase engagement, as they query endogenous kinases from cell extracts (Medard et al., 2015). However, such methods require disruption of plasma membrane integrity, and therefore dilution of the key cellular cofactors (such as ATP) that are known to influence cellular potency. The cellular thermal shift assay (CETSA) represents a significant advancement for intact cell engagement studies, but does not assess engagement under equilibrium conditions nor provide quantitation of target occupancy or affinity (Martinez Molina et al., 2013). CETSA is also reportedly prone to a number of noteworthy false-negative results for multikinase inhibitors, limiting its utility in the context of broad kinome profiling (Savitski et al., 2014). More recent MS-based methods using cell-permeable kinase affinity probes that function in the presence of endogenous ATP offer a more compelling advancement for live-cell profiling (Zhao et al., 2017). However, the formation of a covalent adduct between the reporter probe and the target protein is required prior to lytic analysis via MS. Consequently, such approaches do not allow a quantitative assessment of kinase engagement under a thermodynamic equilibrium with the test molecule. Ideally, methods for quantifying kinase target engagement should be performed within intact cells and offer a quantitative measure of fractional occupancy and affinity for the compound of interest.

Here we report the outcome of the first broad-spectrum, equilibrium-based analysis of inhibitor occupancy in living cells, without dissolution of the cell membrane. Our approach utilizes an energy transfer technique (NanoBRET) that reports on kinase engagement in real time, and enables quantitative inhibitor profiling with a simplified work flow for 178 kinases including over 40 integral membrane receptors. Broad kinome profiling of clinically relevant kinase inhibitors in live cells provides a mechanistic framework for the impact of microenvironmental ATP concentrations on cellular target engagement for human kinases.

RESULTS AND DISCUSSION

Quantitative Measurements of Kinase Engagement for Type I–IV Inhibitors in Live Cells

Traditional biochemical assays provide a convenient and robust platform for quantitation of binding or enzymatic inhibition, but do not allow for assessments of compound permeability or affinity in the presence of cellular factors that may influence potency (Smyth and Collins, 2009). To develop a broad platform to measure kinase engagement in live cells, we exploited a recently described technique using bioluminescence resonance energy transfer (NanoBRET) as a proximity-based measure of compound binding (Robers et al., 2015). In brief, a quantitative analysis can be performed in live cells via energy transfer between a 19-kDa luciferase (NanoLuc, Nluc)-tagged target protein and a cell-permeable fluorescent energy transfer probe introduced to the culture medium (Figure 1). Compound binding results in competitive displacement of the probe and a loss of energy transfer in live cells. The measurement is exclusive to only intact cells, based on the addition of a cell-impermeable inhibitor of Nluc (which gates out signals originating from targets in dead cells or cell culture debris) (Walker et al., 2017). Previously, this technique has successfully been used to quantify compound engagement over a number of intracellular target proteins, and is compatible with scalable work flows and microplate-compatible instrumentation (Robers et al., 2015; Forster et al., 2016).

To enable a kinome-wide capability, a number of cell-permeable, fluorescent energy transfer probes were developed based on a diverse set of ATP-competitive kinase inhibitors (Figure 2A). Type I kinase inhibitors generally bind with minimal conformational bias (Wodicka et al., 2010; Garuti et al., 2010), and were thus selected as inhibitor scaffolds for the development of broad-coverage energy transfer probes. With the exception of probe **4**, all probes were all derived from type I kinase inhibitors (Figure 2A).

Target specificity is achieved based on the dependence of proximity between the Nluc and the energy transfer probe.

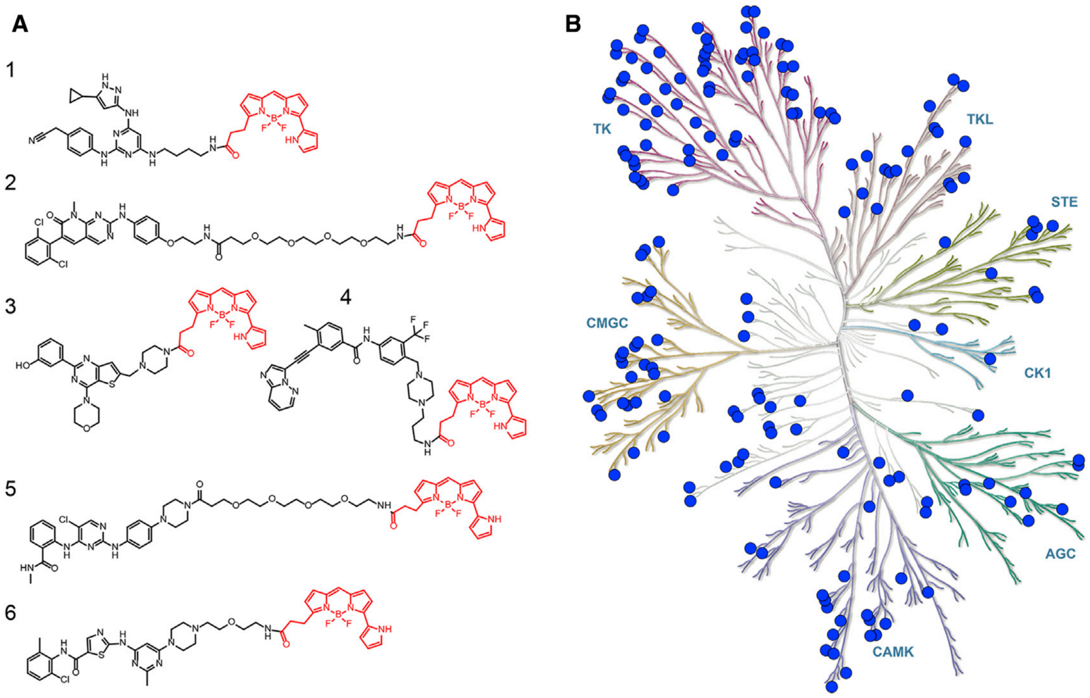

Figure 2. Broad-Coverage, Cell-Permeable Energy Transfer Probes Enable Target Engagement Assays for a Diverse Portion of the Kinome

(A) Cell-permeable energy transfer probes for kinase profiling. **1** is derived from the APY-24 inhibitor core (Gower et al., 2016), **2** is derived from PD-166285 (Drewry et al., 2017), **3** is a derivative of compound **5** from Nacht et al. (2013), **4** is derived from ponatinib (Fauster et al., 2015), **5** is derived from CTx-0294885 (Zhang et al., 2013), and **6** is derived from dasatinib (Johnson et al., 2010).

(B) Dendrogram-based illustration of the broad-kinase coverage using cell-permeable energy transfer probes. Images were generated courtesy of Cell Signaling Technologies.

Each probe was characterized in cells via energy transfer, using a library of plasmid DNAs encoding N- or C-terminal genetic fusions of full-length kinases with Nluc. To determine engagement over this kinase library, we determined the apparent affinity of each energy transfer probe by titration onto live HEK293 cells expressing full-length kinase/Nluc fusion protein. As shown in Figure 2B (and Table S1), currently 178 full-length kinases can be queried using this energy technique, with good representation across the kinome subfamilies (Figures 2, 3, 4, and S1). This approach is even well suited for large, multi-domain proteins such as LRRK2 (286 kDa) and IRE1α/ERN1 (Table S1). Forty full-length receptor tyrosine kinase (RTK) assays were achieved, representing the broadest cellular target engagement approach for these integral membrane receptors. Bioluminescence imaging analysis (Figure S2) confirmed appropriate subcellular localization for kinases used in this study, including DDR1 (plasma membrane), IRE1α (ER), and p38α/MAPK14 (cytoplasm) (Li et al., 2010; Wang et al., 2009; Wood et al., 2009). The ability to interrogate kinase engagement in such complex cellular compartments may offer a general advantage over MS approaches, which are often more limited to highly soluble or abundant kinases (Savitski et al., 2014). In total, 178 cellular kinases were successfully queried with only six energy transfer probes. However, not all kinases tested against the probe set yielded robust assay signals. With increasing characterization of publicly available kinase inhibitor scaffolds (Elkins et al., 2016), it should be possible in the future to develop a more diverse set of probes that span a larger fraction of the kinome.

Use of reversible, ATP-competitive energy transfer probes should enable a quantitative measure of intracellular target occupancy. As demonstrated in Figure 3, the Cheng-Prusoff relationship between the reporter and the unlabeled molecule can be established in live cells, which allows determination of the apparent dissociation constant (K_D) for a test compound (Newton et al., 2008). For DDR1 as an example, the linearized Cheng-Prusoff analysis (Newton et al., 2008) yielded an apparent K_D of 0.5 nM for dasatinib in live HEK293 cells. At energy transfer probe concentrations at or below K_D, this technique should serve as measure of apparent occupancy for the selected kinase in live cells (Cheng and Prusoff, 1973; Newton et al., 2008), provided that the expression level of the target kinase does not impact the measured occupancy values. In cell culture, intracellular target engagement potency (i.e., apparent K_D) should be independent of intracellular kinase concentration, provided that the kinase levels are sufficiently low (Knight and Shokat, 2005). We established that our target engagement results measured with transfected cells remained constant over a range of expression levels, and were similar to those results measured with a target kinase expressed from an endogenous genetic locus. Using the nuclear kinase CDK2 as an example (Hiromura et al., 2002), we were able to titrate expression to levels flanking that of a homozygous cell line engineered to express Nluc as a fusion with CDK2 protein from an endogenous genetic locus (knocked

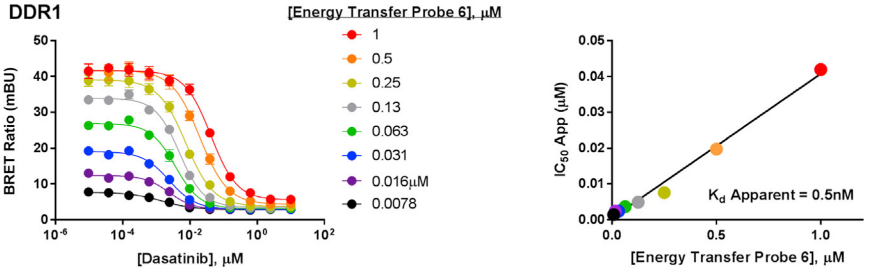

Figure 3. Demonstration of Quantitative Analysis of Cellular Target Engagement Using Energy Transfer

Quantitative analysis can be performed with BRET, using the linearized Cheng-Prusoff relationship (Newton et al., 2008) for DDR1 kinase in live HEK293 cells using probe **6** (left). Linear regression analysis of test compound IC_{50} versus energy transfer probe concentration allows for a determination of apparent K_D from the y intercept (right). Results are the mean ± SE of three independent experiments (n = 3).

in via CRISPR/Cas9). In transfected cells, target engagement results were nearly identical over this broad expression range, and agreed closely with results generated with endogenously expressed CDK2-Nluc protein (Figure S2).

Biochemical analysis of kinase selectivity and affinity may fail to predict engagement in live cells (Smyth and Collins, 2009; Knight and Shokat, 2005; Zhao et al., 2017). For the development of kinase chemical probes for cell biology research, kinase inhibitors must display sufficient intracellular selectivity at the intended target (Arrowsmith et al., 2015). We applied the energy transfer technique to query affinity and selectivity of the novel chemical probe **6j** (Figure S3), a putative selective inhibitor for DDR1 (Wang et al., 2016). In biochemical measurements using the truncated domain of DDR1, **6j** demonstrates 20-fold selectivity over DDR2 (Wang et al., 2016). However, as shown in Figure S3, this selectivity index was considerably lower in cells, suggesting that phenotypic responses may arise from inhibition of a combination of both DDR1 and DDR2. These results further indicate that intracellular kinase selectivity for a given chemical probe may deviate from biochemical predictions.

For large, multidomain kinases, intracellular energy transfer method should address many of the shortcomings of biochemical assay techniques traditionally used to characterize inhibitor selectivity. For example, the reliance on segregated kinase domains in biochemical formats has been shown to limit the ability to detect broad classes of ATP-competitive inhibitors that may bind outside the ATP pocket or exhibit conformation-state-dependent binding (Smyth and Collins, 2009). For many kinases (e.g., RTKs), isolation of full-length kinase for biochemical analysis may be problematic.

To demonstrate the broad applicability of the energy transfer assay for quantitation of engagement for kinase inhibitors utilizing diverse binding mechanisms, we performed equilibrium-based analysis of engagement for a set of full-length kinase targets in live cells. This target panel was composed of ABL, PIK3CA (PI3Kα), BTK, RIPK1, LIMK1, and LCK. As shown in Figure 4 (and Figure S1), affinity profiles were successfully established at each kinase. Use of full-length kinases allowed us to query the engagement of various types of ATP-competitive inhibitors, which bind sites within, adjacent to, or distal to the nucleotide binding pocket (type I/II, III, and IV, respectively). Analysis of RIPK1 engagement revealed binding of various type I inhibitors, as well as the type II inhibitor ponatinib and the type III inhibitor necrostatin-1 (nec-1). Similarly, we successfully measured engagement of inhibitors at LIMK1 that utilize diverse mechanisms. Binding of both the type I inhibitor (LIMKi3) and the type III inhibitor TH251 was detected in live cells (Ross-Macdonald et al., 2008; Kashima et al., 2017). For ABL kinase, engagement of type I inhibitors (dasatinib), type II inhibitors (imatinib, ponatinib) and the type IV inhibitor GNF-2 were successfully measured. GNF-2 engages ABL kinase in the myristoyl binding pocket, located distally to the ATP binding site (Kim et al., 2014; Choi et al., 2010; Zhang et al., 2010). Only partial displacement of GNF-2 was observed against the energy transfer probe, possibly due to the unique binding mode of GNF-2 at ABL. It has been previously established that both nec-1 and GNF-2 are functionally competitive with ATP (Iacob et al., 2011; Degterev et al., 2008), consistent with our ability to detect their kinase interactions using type I, ATP-competitive energy transfer probes. These results support use of the energy transfer technique for querying engagement of compounds that bind via mechanisms dissimilar from that of the energy transfer probe, as long as the binding of the compound and the probe are mutually exclusive.

For energy transfer probes derived from type I kinase inhibitor scaffolds, compound potencies should be generally unaffected by the probe chemotype. RIPK1 binds multiple energy transfer probes developed in this study, and therefore served to demonstrate this independence. For the type I inhibitors measured against RIPK1, engagement potency was similar (within 2-fold) when using probe **1** or probe **2** (Figure S3).

Intracellular Target Engagement by Energy Transfer Correlates with Inhibition of Intracellular Kinase Activity

As discussed earlier, measurements of binding or inhibition of kinases in isolation may fail to accurately predict engagement in the complex milieu of live cells (Smyth and Collins, 2009; Cui et al., 2011). To demonstrate that the energy transfer method could establish a functional link between physical binding and modulation of intracellular kinase activity, we performed a correlation analysis between target engagement via energy transfer and kinase function using a more traditional cellular pathway analysis. In the absence of suitable biophysical methods, cellular pathway analysis tools may serve as proxies to infer intracellular target engagement for kinases. For example, substrate phosphorylation analysis has previously served as a measure of intracellular inhibition for the targets of the multikinase inhibitor (R)-crizotinib (Cui et al., 2011). Comparison of cellular phospho-ELISA results versus enzymatic half-maximal inhibitory concentration (IC_{50}) values revealed significant potency offsets for crizotinib for nearly all of the queried kinases (Cui et al., 2011) (Table S2). The cellular offset was most notable for kinase LCK, which demonstrated a >3-log shift in cellular

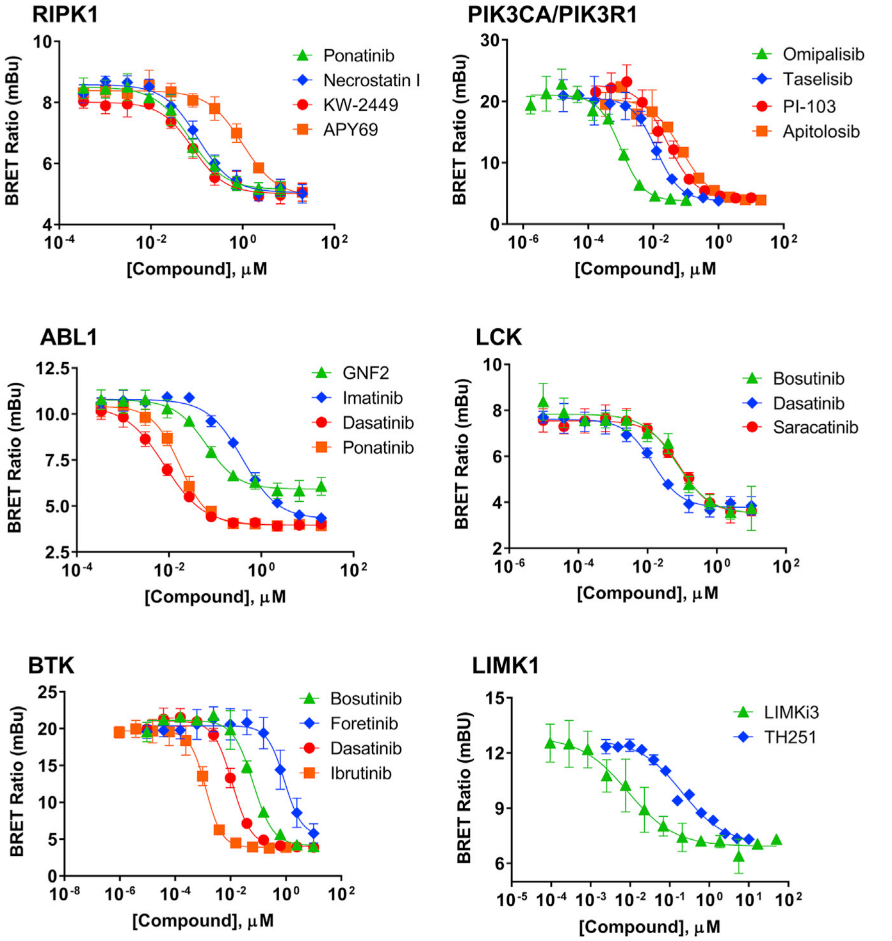

Figure 4. Examples of Intracellular Target Engagement Analysis for Type I, Type II, and Allosteric Kinase Inhibitors against Full-Length Kinases in Live HEK293 Cells
RIPK1, PIK3CA, ABL, LCK, BTK, and LIMK1 used probes **2**, **3**, **6**, **5**, **5**, and **5**, respectively. Individual data points are the mean ± SD of at least three technical replicates. For LIMK1, data are mean ± SE of two independent experiments performed in 384-well format (n = 2). All other experiments were performed at least twice (n = 2), with representative data from independent experimental replicates presented in Figure S1.

potency (3 μM) compared with the reported biochemical value (<1 nM) (Cui et al., 2011).

We used energy transfer to measure intracellular target engagement potencies against the same group of tyrosine kinases analyzed via phospho-ELISA (Cui et al., 2011). Consistent with intracellular phosphorylation analysis, highest engagement potencies were observed for the primary targets MET and ALK, with potencies ranging over approximately three orders of magnitude for the collateral targets (Figures 5A and S3). In contrast to the acellular enzymatic analysis, live-cell engagement analysis in HEK293 cells revealed engagement potencies in close agreement (R^2 = 0.95) with published cellular potency values determined by phospho-ELISA (Figures 5B and S3). These results indicate that kinase engagement measured via energy transfer can be predictive of inhibition of intracellular kinase activity. With the emergence of new genome editing and expression techniques, the BRET method should be expandable to a variety of cell types when a specific cellular context or native expression level is required to accurately reflect cell physiology.

Intracellular Kinase Profiling Reveals Improved Selectivity for Crizotinib and Dasatinib Compared with that Determined from Biochemical Measurements

The potency offset observed between cellular and acellular analysis of kinase inhibition (Cui et al., 2011; Davis et al., 2011) implies that selectivity may be generally improved within cells for promiscuous, ATP-competitive kinase inhibitors. To test this hypothesis, we performed a quantitative, kinome-wide analysis of intracellular occupancy for (R)-crizotinib, and compared our results with published crizotinib profiles using isolated kinases or kinase domains (Davis et al., 2011). Such comprehensive biochemical analyses provide a strong foundation for complementary cellular mechanistic studies (Davis et al., 2011). Our intracellular target engagement analysis included a diversity set comprising 178 full-length kinases spanning each kinase subfamily, including the well-characterized crizotinib targets MET and ALK. Since engagement is dose dependent, we selected a clinically relevant (C_{max}) dose of 1 μM crizotinib for the cellular profiling (Kurata et al., 2015). For each kinase tested, a concentration of energy transfer probe was introduced to the culture medium at an optimized concentration as described in Table S1. Once the appropriate energy transfer probe concentration is determined, the energy transfer technique is easily scaled in a microplate format for single-dose profiling of target occupancy, and can be performed for 178 kinases in a single day (see STAR Methods). Use of plasmid DNA as a source of each cellular kinase fusion circumvents the need for protein purification. Consequently, these features support a relatively simple platform for broad-spectrum profiling in live cells.

Fractional occupancies were determined for each kinase in live cells, and results were compared with published values from previous biochemical binding studies (Davis et al., 2011). Of the 178 kinases in our analysis, only 13 targets were engaged above 50% occupancy at a dose of 1 μM crizotinib (Figure 6A and Table S1). In contrast, previous biochemical analysis of crizotinib suggested that 69 of our queried kinases would be engaged over the same 50% occupancy threshold at 1 μM (Davis et al., 2011). As expected, the kinases MET and ALK were highly occupied by 1 μM crizotinib in cells. However, a number of putative collateral crizotinib targets such as LCK and the pseudokinase EPHB6 were unoccupied in cells despite previous reports of strong biochemical affinity (K_D = 30 nM and

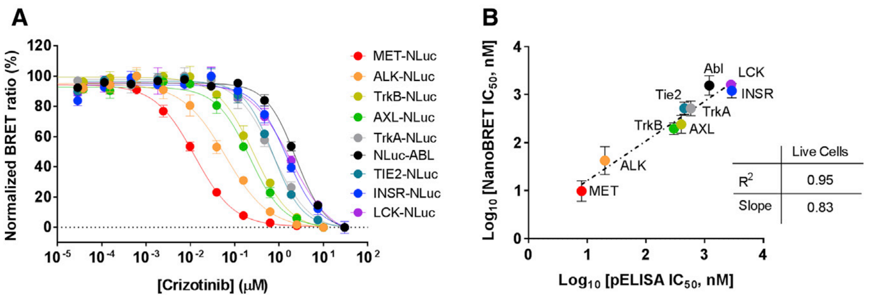

Figure 5. Target Engagement Profiling of Crizotinib in Live Cells Correlates with Reported Intracellular Kinase Inhibition

(A) Representative data from cellular target engagement profiling of crizotinib against various full-length kinases in live HEK293 cells. All kinases were queried using probe **5**, except ABL1, which used probe **6**. Individual data points are the mean ± SD of four technical replicates from experiments performed three times (n = 3).

(B) Correlation of crizotinib apparent affinity in live cells versus published potency for inhibition of intracellular kinase activity via phospho-ELISA (Cui et al., 2011). R^2 = 0.95. Data are the mean ± SE from three independent experiments (n = 3).

6 nM, respectively) (Davis et al., 2011). To establish that the improved spectrum of activity observed in cells was not unique to crizotinib, we also performed a systematic, broad-spectrum profiling of the cellular targets of dasatinib at a clinically relevant dose of 100 nM (Johnson et al., 2010). Consistent with the observations for crizotinib, our analysis of dasatinib revealed improved selectivity compared with previous biochemical profiling results (Figure 6B and Table S1), yielding 22 targets engaged in cells compared with 44 targets engaged biochemically at the 50% occupancy threshold (Davis et al., 2011). Our dasatinib engagement results provided a dataset for comparison with previous work using MS-based profiling approaches, such as those that utilize thermal denaturation or covalent capture. Thermal profiling in live cells failed to identify the BCR-ABL oncoprotein as a direct target of dasatinib (Savitski et al., 2014), whereas energy transfer confirmed high occupancy of ABL at a 100-nM dose. Our analysis also revealed a broader engagement profile compared with a covalent capture probe approach in live Jurkat cells (Zhao et al., 2017), which identified that only six kinases occupied >50% at 100 nM dasatinib. Numerous RTKs were engaged with high occupancy with 100 nM dasatinib in our analysis. It is possible that many of these integral membrane proteins could be expressed at low levels or could be incompatible for target capture, and therefore are undetectable by MS. These factors may lead to the narrower spectrum of engagement compared with our results.

Our cellular profiling results were highly reproducible over the kinase diversity set (Figure S4), supporting the energy transfer technique as a robust approach to live-cell kinase profiling. Moreover, these results support a cellular selectivity mechanism that may be a general feature of ATP-competitive kinase inhibitors, and underscore the value of broad kinase profiling in live cells for determination of inhibitor selectivity.

Mechanistic Analysis of Kinase Engagement Reveals the Effect of Local Cellular ATP on Drug Potency

The occupancy profile and pronounced potency offset observed for crizotinib suggests that endogenous ATP could be contributing to target engagement interference. However, this interpretation is confounded by a number of interdependent factors resident to cells that must be considered. First, ATP interference is presumably influenced by the intrinsic affinity of ATP for each kinase, representing a key variable for cellular target engagement. Moreover, the K_M of ATP is known to be heavily influenced by kinase activation state, which may exist in a dynamic equilibrium in cells (Wodicka et al., 2010; Till et al., 2002). Finally, local ATP concentrations may vary depending on the cellular localization of the target, suggesting that potency shifts may be challenging to functionally deconvolute in a cellular environment (Niki et al., 1989) and difficult to simulate biochemically.

Using the energy transfer technique, we performed a mechanistic analysis of ATP interference on cellular kinase engagement. As shown in Figure 6A, the cellular engagement profile for crizotinib revealed a number of putative, high-affinity targets that were surprisingly unoccupied in cells. For example, the affinity of the tyrosine kinase LCK and pseudokinase EPHB6 for crizotinib are reported to be 30 nM and 6 nM, respectively, in biochemical analyses (Davis et al., 2011), but both unexpectedly show low cellular occupancy (<10%) at a dose of 1 μM. Conversely, a number of relatively poor-affinity crizotinib targets such as MuSK (230 nM), CASK (140 nM), and TYRO3 (800 nM) displayed high cellular occupancy under these conditions (Davis et al., 2011). This discordance suggests that a cellular factor such as ATP may represent a key variable influencing cellular potency for these kinases. We therefore attempted to determine the relative influences of the cosubstrate ATP on engagement profiles for these individual kinases and pseudokinases.

To investigate this discordance between cellular and acellular engagement, we assessed the influence of ATP on the engagement profile for crizotinib in live cells. This was accomplished by performing live-cell target engagement analysis at physiological ATP levels, along with a similar analysis performed in cells depleted of ATP. To modulate intracellular ATP concentrations, we performed our analysis with live cells depleted of intracellular ATP through inhibition of glycolysis and mitochondrial ATP synthesis. This approach preserves plasma membrane integrity (assessed by exclusion of a cell-impermeable DNA dye) and cellular architecture, but lowers intracellular ATP levels up to 20-fold (Figure S5) (Swiss et al., 2013) over the duration of the assay (assessed by quantitation of total [ATP]). As shown in Figure 7 (and Figure S6), ATP-depleted cells with full membrane integrity showed an enhanced (i.e., left-shifted) engagement profile for crizotinib for the kinases LCK and EPHB6, suggesting interference from cellular ATP. Moreover, analysis using permeabilized cells (more severely depleted of ATP) resulted in a more pronounced left-shifted crizotinib affinity for these kinases (Figures 7 and S6). The K_D for crizotinib obtained using permeabilized cells (14 nM and 70 nM for EPHB6 and LCK, respectively) were in close agreement with those reported from biochemical analysis, suggesting that the Nluc tag has minimal influence on the binding behavior

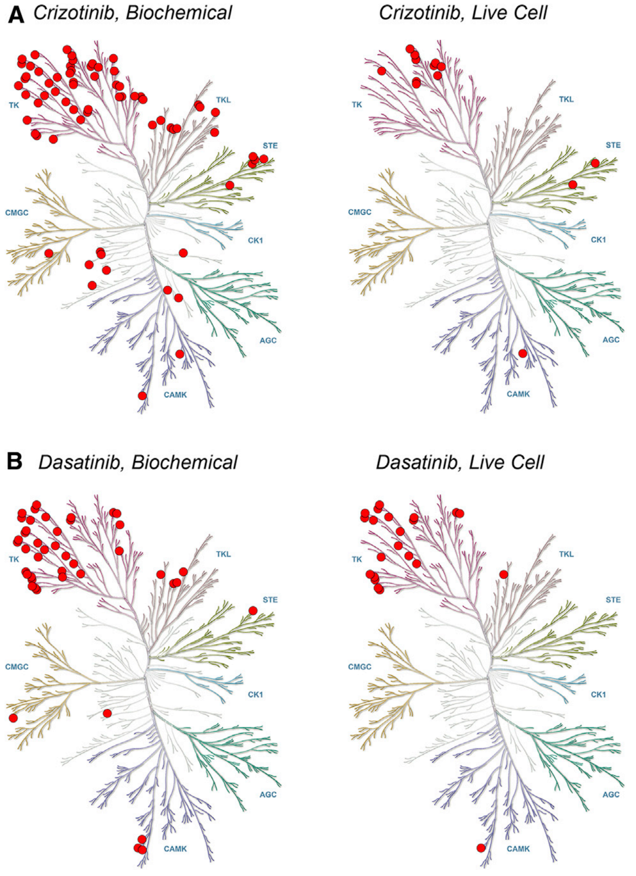

Figure 6. Cellular Profiling Reveals Improved Selectivity for the Multikinase Inhibitors (R)-Crizotinib and Dasatinib

(A) Dendrogram-based comparison of published target occupancy with 1 μM crizotinib using recombinant kinases assayed biochemically (Davis et al., 2011) versus intracellular kinases in HEK293 cells (right). Each mark represents a target engaged at or above a minimum 50% occupancy threshold.

(B) Dendrogram-based comparison of target occupancy with 100 nM dasatinib using kinases assayed biochemically (Davis et al., 2011) (left) versus intracellular kinases in HEK293 cells (right). Data are the mean of two independent experiments (n = 2). Images courtesy of Cell Signaling Technologies.

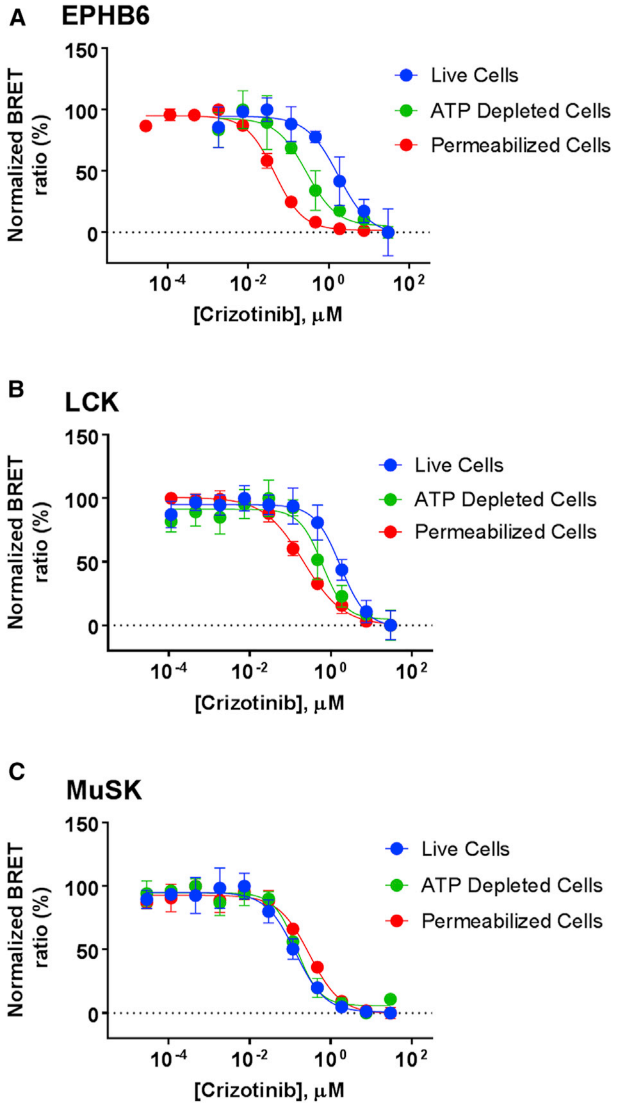

Figure 7. Mechanistic Analysis of ATP Interference on Target Occupancy in Live Cells

EPHB6 was queried using probe **6**, while LCK and MuSK where queried using probe **5**, all at probe concentrations optimized for each specific assay condition (live, ATP-depleted, and permeabilized; see Figure S4). Depletion of intracellular ATP in live cells or by permeabilization results in increased engagement potency of (R)-crizotinib against the kinase LCK (A) and the pseudokinase EPHB6 (B). (C) MuSK kinase shows negligible interference from intracellular ATP, thus providing a mechanistic explanation for the relative occupancies of these kinases with 1 μM crizotinib in live-cell profiling experiments. Results are the mean ± SE of three independent experiments (n = 3).

of these kinase targets and that the energy transfer approach in a permeabilized cell format provides a suitable biochemical proxy for determination of compound affinity in full-length proteins (Figures S6 and S7). Moreover, we were also able to confirm ATP affinity for both LCK and EPHB6 by titration of exogenous nucleotide in the permeabilized cell format (Figures S6 and S7).

Compared with the strong affinity targets LCK and EPHB6, the reported biochemical affinity of MuSK for crizotinib is relatively weak. Thus, the high intracellular occupancy of MuSK with 1 μM crizotinib was unexpected. To address this discordance, we investigated whether MuSK was similarly competitive with cellular ATP. In contrast to cellular target engagement results with LCK and EPHB6, MuSK demonstrated negligible interference with ATP, as measured in all ATP-depleted conditions (Figures 7 and S6). Corroborating this result, MuSK showed no observed binding to exogenous ATP titrated into permeabilized cells (Figures S6 and S7). It is noteworthy that the reported K_M of ATP for MuSK is highly dependent on kinase activation state (14 μM for active kinase compared with >3,000 μM for inactive kinase), raising the possibility of activity dependence on intracellular target engagement for MuSK (Till et al., 2002). Our results support that MuSK (along with a number of other kinases) may exist in an inactive state that is relatively insensitive to competition with endogenous ATP, at least under unstimulated conditions. Furthermore, these results indicate that reported ATP K_M values using isolated kinase domains may not always reflect cellular physiology and the dynamics of intracellular kinase

activation state. However, as this analysis was performed in HEK293 cells, it may be challenging to assess activation-state-dependent binding for all queried kinases. Assessments of MuSK engagement in muscle cells could make it possible to investigate engagement potency under active versus inactive kinase states. As new genome editing techniques progress, it should be feasible to introduce this energy transfer technique into such physiologically relevant cell models when required. Our successful use of CRISPR/Cas9 for the engineering of endogenous CDK2 for the energy transfer technique (Figure S2) supports this as a feasible direction.

SIGNIFICANCE

In this study, we utilized a live-cell energy transfer technique to perform a broad-coverage, quantitative analysis of intracellular kinase target engagement. Unlike existing methods, this technique is compatible with analysis under equilibrium conditions, thus allowing for determination of fractional target occupancy and affinity for various classes of ATP-competitive inhibitors in live cells. Broad-spectrum analysis of target occupancy uncovered an improved selectivity profile for clinically relevant multikinase inhibitors. A systematic analysis of engagement affinity in ATP-depleted cells allowed us to examine the influence of local cellular ATP concentrations on the putative targets of the multikinase inhibitor crizotinib. Moreover, the target engagement results obtained for crizotinib translated well with published cellular potency values measured via phospho-ELISA. These findings support cellular target engagement profiling as a more accurate predictor of intracellular drug selectivity compared with standard biochemical approaches, offering a potentially valuable tool to guide medicinal chemistry during the development of chemical probes or clinical drug candidates. With a quantitative and accurate kinase target engagement technique, cellular target engagement profiling should be performed as standard practice for human kinases.

STAR★METHODS

Detailed methods are provided in the online version of this paper and include the following:

- KEY RESOURCES TABLE
- CONTACT FOR REAGENT AND RESOURCE SHARING
- EXPERIMENTAL MODEL AND SUBJECT DETAILS
- METHOD DETAILS
  - Cell Transfections and BRET Measurements
  - Cellular ATP Depletion
  - Endogenous Tagging Using CRISPR/Cas9
  - Bioluminescence Imaging
  - Chemical Synthesis
- QUANTIFICATION AND STATISTICAL ANALYSIS

SUPPLEMENTAL INFORMATION

Supplemental Information includes seven figures and two tables and can be found with this article online at https://doi.org/10.1016/j.chembiol.2017.10.010.

AUTHOR CONTRIBUTIONS

J.D.V. and M.B.R. designed experiments and wrote the paper. J.D.V., C.R.C., J.W., C.A.Z., J.R.H., M.R.I., K.Z., T.M., T.A.K., K.G.H., R.F.O., M.S., P.O., M.C., C.I.W., B.-T.B., T.H., C.G., K.D., D.H.D., K.V.M.H., T.M.W., S.K., S.M., P.L.M., F.F., K.V.W., M.B.R. contributed to the design and/or execution of experiments.

ACKNOWLEDGMENTS

The authors thank Sergiy Levin for chemistry advice and Ethan Strauss for his advice regarding kinase bioinformatics. The SGC is a registered charity (number 1097737) that receives funds from AbbVie, Bayer Pharma, Boehringer Ingelheim, Canada Foundation for Innovation, Eshelman Institute for Innovation, Genome Canada through Ontario Genomics Institute (OGI-055), Innovative Medicines Initiative (EU/EFPIA) (ULTRA-DD grant no. 115766), Janssen, Merck, MSD, Novartis Pharma, Ontario Ministry of Research, Innovation and Science (MRIS), Pfizer, São Paulo Research Foundation - FAPESP, Takeda, and the Wellcome Trust (grant no. 106169/ZZ14/Z). J.D.V., C.R.C., J.W., C.A.Z., J.R.H., M.R.I., K.Z., T.M., M.S., P.O., T.A.K., K.G.H., R.F.O., M.C., P.L.M., F.F., K.V.W., and M.B.R. are employees of Promega.

Received: June 21, 2017
Revised: August 24, 2017
Accepted: October 24, 2017
Published: November 22, 2017

REFERENCES

Arrowsmith, C.H., Audia, J.E., Austin, C., Baell, J., Bennett, J., Blagg, J., Bountra, C., Brennan, P.E., Brown, P.J., Bunnage, M.E., et al. (2015). The promise and peril of chemical probes. Nat. Chem. Biol. *11*, 536–541.

Becher, I., Savitski, M.M., Savitski, M.F., Hopf, C., Bantscheff, M., and Drewes, G. (2013). Affinity profiling of the cellular kinome for the nucleotide cofactors ATP, ADP, and GTP. ACS Chem. Biol. *8*, 599–607.

Cheng, Y., and Prusoff, W.H. (1973). Relationship between the inhibition constant (K1) and the concentration of inhibitor which causes 50 per cent inhibition (I50) of an enzymatic reaction. Biochem. Pharmacol. *22*, 3099–3108.

Choi, H.G., Ren, P., Adrian, F., Sun, F., Lee, H.S., Wang, X., Ding, Q., Zhang, G., Xie, Y., Zhang, J., et al. (2010). A type-II kinase inhibitor capable of inhibiting the T315I "gatekeeper" mutant of Bcr-Abl. J. Med. Chem. *53*, 5439–5448.

Cui, J.J., Tran-Dube, M., Shen, H., Nambu, M., Kung, P.P., Pairish, M., Jia, L., Meng, J., Funk, L., Botrous, I., et al. (2011). Structure based drug design of crizotinib (PF-02341066), a potent and selective dual inhibitor of mesenchymal-epithelial transition factor (c-MET) kinase and anaplastic lymphoma kinase (ALK). J. Med. Chem. *54*, 6342–6363.

Davis, M.I., Hunt, J.P., Herrgard, S., Ciceri, P., Wodicka, L.M., Pallares, G., Hocker, M., Treiber, D.K., and Zarrinkar, P.P. (2011). Comprehensive analysis of kinase inhibitor selectivity. Nat. Biotechnol. *29*, 1046–1051.

Degterev, A., Hitomi, J., Germscheid, M., Ch'en, I.L., Korkina, O., Teng, X., Abbott, D., Cuny, G.D., Yuan, C., Wagner, G., et al. (2008). Identification of RIP1 kinase as a specific cellular target of necrostatins. Nat. Chem. Biol. *4*, 313–321.

Drewry, D.H., Wells, C.I., Andrews, D.M., Angell, R., Al-Ali, H., Axtman, A.D., Capuzzi, S.J., Elkins, J.M., Ettmayer, P., Frederiksen, M., et al. (2017). Progress towards a public chemogenomic set for protein kinases and a call for contributions. PLoS One *12*, e0181585.

Elkins, J.M., Fedele, V., Szklarz, M., Abdul Azeez, K.R., Salah, E., Mikolajczyk, J., Romanov, S., Sepetov, N., Huang, X.P., et al. (2016). Comprehensive characterization of the published kinase inhibitor set. Nat. Biotechnol. *34*, 95–103.

Fauster, A., Rebsamen, M., Huber, K.V., Bigenzahn, J.W., Stukalov, A., Lardeau, C.H., Scorzoni, S., Bruckner, M., Gridling, M., Parapatics, K., et al. (2015). A cellular screen identifies ponatinib and pazopanib as inhibitors of necroptosis. Cell Death Dis. *6*, e1767.

Forster, M., Chaikuad, A., Bauer, S.M., Holstein, J., Robers, M.B., Corona, C.R., Gehringer, M., Pfaffenrot, E., Ghoreschi, K., Knapp, S., and Laufer,

S.A. (2016). Selective JAK3 inhibitors with a covalent reversible binding mode targeting a new induced fit binding pocket. Cell Chem Biol. *23*, 1335–1340.

Garuti, L., Roberti, M., and Bottegoni, G. (2010). Non-ATP competitive protein kinase inhibitors. Curr. Med. Chem. *17*, 2804–2821.

Gower, C.M., Thomas, J.R., Harrington, E., Murphy, J., Chang, M.E., Cornella-Taracido, I., Jain, R.K., Schirle, M., and Maly, D.J. (2016). Conversion of a single polypharmacological agent into selective bivalent inhibitors of intracellular kinase activity. ACS Chem. Biol. *11*, 121–131.

Hiromura, K., Pippin, J.W., Blonski, M.J., Roberts, J.M., and Shankland, S.J. (2002). The subcellular localization of cyclin dependent kinase 2 determines the fate of mesangial cells: role in apoptosis and proliferation. Oncogene *21*, 1750–1758.

Hulme, E.C., and Trevethick, M.A. (2010). Ligand binding assays at equilibrium: validation and interpretation. Br. J. Pharmacol. *161*, 1219–1237.

Iacob, R.E., Zhang, J., Gray, N.S., and Engen, J.R. (2011). Allosteric interactions between the myristate- and ATP-site of the Abl kinase. PLoS One *6*, e15929.

Imamura, H., Nhat, K.P., Togawa, H., Saito, K., Iino, R., Kato-Yamada, Y., Nagai, T., and Noji, H. (2009). Visualization of ATP levels inside single living cells with fluorescence resonance energy transfer-based genetically encoded indicators. Proc. Natl. Acad. Sci. USA *106*, 15651–15656.

Johnson, F.M., Bekele, B.N., Feng, L., Wistuba, I., Tang, X.M., Tran, H.T., Erasmus, J.J., Hwang, L.L., Takebe, N., Blumenschein, G.R., et al. (2010). Phase II study of dasatinib in patients with advanced non-small-cell lung cancer. J. Clin. Oncol. *28*, 4609–4615.

Kashima, R., Redmond, P.L., Ghatpande, P., Roy, S., Kornberg, T.B., Hanke, T., Knapp, S., Lagna, G., and Hata, A. (2017). Hyperactive locomotion in a *Drosophila* model is a functional readout for the synaptic abnormalities underlying fragile X syndrome. Sci. Signal. *10*, https://doi.org/10.1126/scisignal.aai8133.

Kim, H.J., Yoon, H.J., Choi, J.Y., Lee, I.K., and Kim, S.Y. (2014). The tyrosine kinase inhibitor GNF-2 suppresses osteoclast formation and activity. J. Leukoc. Biol. *95*, 337–345.

Klutchko, S.R., Hamby, J.M., Boschelli, D.H., Wu, Z., Kraker, A.J., Amar, A.M., Hartl, B.G., Shen, C., Klohs, W.D., Steinkampf, R.W., et al. (1998). 2-Substituted aminopyrido[2,3-d]pyrimidin-7(8H)-ones. structure-activity relationships against selected tyrosine kinases and in vitro and in vivo anticancer activity. J. Med. Chem. *41*, 3276–3292.

Knight, Z.A., and Shokat, K.M. (2005). Features of selective kinase inhibitors. Chem. Biol. *12*, 621–637.

Kurata, Y., Miyauchi, N., Suno, M., Ito, T., Sendo, T., and Kiura, K. (2015). Correlation of plasma crizotinib trough concentration with adverse events in patients with anaplastic lymphoma kinase positive non-small-cell lung cancer. J. Pharm. Health Care Sci. *1*, 8.

Li, H., Korennykh, A.V., Behrman, S.L., and Walter, P. (2010). Mammalian endoplasmic reticulum stress sensor IRE1 signals by dynamic clustering. Proc. Natl. Acad. Sci. USA *107*, 16113–16118.

Marroquin, L.D., Hynes, J., Dykens, J.A., Jamieson, J.D., and Will, Y. (2007). Circumventing the Crabtree effect: replacing media glucose with galactose increases susceptibility of HepG2 cells to mitochondrial toxicants. Toxicol. Sci. *97*, 539–547.

Martinez Molina, D., Jafari, R., Ignatushchenko, M., Seki, T., Larsson, E.A., Dan, C., Sreekumar, L., Cao, Y., and Nordlund, P. (2013). Monitoring drug target engagement in cells and tissues using the cellular thermal shift assay. Science *341*, 84–87.

Medard, G., Pachl, F., Ruprecht, B., Klaeger, S., Heinzlmeir, S., Helm, D., Qiao, H., Ku, X., Wilhelm, M., Kuehne, T., et al. (2015). Optimized chemical proteomics assay for kinase inhibitor profiling. J. Proteome Res. *14*, 1574–1586.

Nacht, M., Qiao, L., Sheets, M.P., St Martin, T., Labenski, M., Mazdiyasni, H., Karp, R., Zhu, Z., Chaturvedi, P., Bhavsar, D., et al. (2013). Discovery of a potent and isoform-selective targeted covalent inhibitor of the lipid kinase PI3Kα. J. Med. Chem. *56*, 712–721.

Newton, P., Harrison, P., and Clulow, S. (2008). A novel method for determination of the affinity of protein:protein interactions in homogeneous assays. J. Biomol. Screen. *13*, 674–682.

Niki, I., Ashcroft, F.M., and Ashcroft, S.J. (1989). The dependence on intracellular ATP concentration of ATP-sensitive K-channels and of Na,K-ATPase in intact HIT-T15 beta-cells. FEBS Lett. *257*, 361–364.

Robers, M.B., Dart, M.L., Woodroofe, C.C., Zimprich, C.A., Kirkland, T.A., Machleidt, T., Kupcho, K.R., Levin, S., Hartnett, J.R., Zimmerman, K., et al. (2015). Target engagement and drug residence time can be observed in living cells with BRET. Nat. Commun. *6*, 10091.

Ross-Macdonald, P., De Silva, H., Guo, Q., Xiao, H., Hung, C.Y., Penhallow, B., Markwalder, J., He, L., Attar, R.M., Lin, T.A., et al. (2008). Identification of a nonkinase target mediating cytotoxicity of novel kinase inhibitors. Mol. Cancer Ther. *7*, 3490–3498.

Savitski, M.M., Reinhard, F.B., Franken, H., Werner, T., Savitski, M.F., Eberhard, D., Martinez Molina, D., Jafari, R., Dovega, R.B., Klaeger, S., et al. (2014). Tracking cancer drugs in living cells by thermal profiling of the proteome. Science *346*, 1255784.

Smyth, L.A., and Collins, I. (2009). Measuring and interpreting the selectivity of protein kinase inhibitors. J. Chem. Biol. *2*, 131–151.

Statsuk, A.V., Maly, D.J., Seeliger, M.A., Fabian, M.A., Biggs, W.H., 3rd, Lockhart, D.J., Zarrinkar, P.P., Kuriyan, J., and Shokat, K.M. (2008). Tuning a three-component reaction for trapping kinase substrate complexes. J. Am. Chem. Soc. *130*, 17568–17574.

Swiss, R., Niles, A., Cali, J.J., Nadanaciva, S., and Will, Y. (2013). Validation of a HTS-amenable assay to detect drug-induced mitochondrial toxicity in the absence and presence of cell death. Toxicol. In Vitro *27*, 1789–1797.

Till, J.H., Becerra, M., Watty, A., Lu, Y., Ma, Y., Neubert, T.A., Burden, S.J., and Hubbard, S.R. (2002). Crystal structure of the MuSK tyrosine kinase: insights into receptor autoregulation. Structure *10*, 1187–1196.

Walker, J.R., Hall, M.P., Zimprich, C.A., Robers, M.B., Duellman, S.J., Machleidt, T., Rodriguez, J., and Zhou, W. (2017). Highly potent cell-permeable and impermeable nanoluc luciferase inhibitors. ACS Chem. Biol. *12*, 1028–1037.

Wang, C.Z., Yeh, Y.C., and Tang, M.J. (2009). DDR1/E-cadherin complex regulates the activation of DDR1 and cell spreading. Am. J. Physiol. Cell Physiol. *297*, C419–C429.

Wang, Z., Bian, H., Bartual, S.G., Du, W., Luo, J., Zhao, H., Zhang, S., Mo, C., Zhou, Y., Xu, Y., et al. (2016). Structure-based design of tetrahydroisoquinoline-7-carboxamides as selective discoidin domain receptor 1 (DDR1). Inhibitors. J. Med. Chem. *59*, 5911–5916.

Wodicka, L.M., Ciceri, P., Davis, M.I., Hunt, J.P., Floyd, M., Salerno, S., Hua, X.H., Ford, J.M., Armstrong, R.C., Zarrinkar, P.P., and Treiber, D.K. (2010). Activation state-dependent binding of small molecule kinase inhibitors: structural insights from biochemistry. Chem. Biol. *17*, 1241–1249.

Wood, C.D., Thornton, T.M., Sabio, G., Davis, R.A., and Rincon, M. (2009). Nuclear localization of p38 MAPK in response to DNA damage. Int. J. Biol. Sci. *5*, 428–437.

Wu, P., Nielsen, T.E., and Clausen, M.H. (2016). Small-molecule kinase inhibitors: an analysis of FDA-approved drugs. Drug Discov. Today *21*, 5–10.

Zhang, J., Adrian, F.J., Jahnke, W., Cowan-Jacob, S.W., Li, A.G., Iacob, R.E., Sim, T., Powers, J., Dierks, C., Sun, F., et al. (2010). Targeting Bcr-Abl by combining allosteric with ATP-binding-site inhibitors. Nature *463*, 501–506.

Zhang, L., Holmes, I.P., Hochgrafe, F., Walker, S.R., Ali, N.A., Humphrey, E.S., Wu, J., De Silva, M., Kersten, W.J., Connor, T., et al. (2013). Characterization of the novel broad-spectrum kinase inhibitor CTx-0294885 as an affinity reagent for mass spectrometry-based kinome profiling. J. Proteome Res. *12*, 3104–3116.

Zhao, Q., Ouyang, X., Wan, X., Gajiwala, K.S., Kath, J.C., Jones, L.H., Burlingame, A.L., and Taunton, J. (2017). Broad-spectrum kinase profiling in live cells with lysine-targeted sulfonyl fluoride probes. J. Am. Chem. Soc. *139*, 680–685.

STAR★METHODS

KEY RESOURCES TABLE

REAGENT or RESOURCE	SOURCE	IDENTIFIER
Antibodies		
CDK2 (78B2) Rabbit mAb antibody	Cell Signaling Technologies	CAT# 2546S; RRID: AB_10695594
Chemicals, Peptides, and Recombinant Proteins		
Cas9 protein (HIS-sNLS-Myc-spCas9)	Aldevron	N/A
High fidelity DNA polymerase Q5	New England Biolabs	CAT# M0494S
GoTaq Hot Start Green Master Mix	Promega	CAT# M5123
SuperSignal West Pico Detection Kit	Thermo	CAT# 34578
NanoBRET 590 SE	Promega	CAT# CS189404
FuGENE HD	Promega	CAT# E2311
Opti-MEM without phenol red	Gibco	CAT# 11058021
Tracer Dilution Buffer	Promega	CAT# N219A
NanoBRET NanoGlo Substrate	Promega	CAT# N157A
Extracellular NanoLuc Inhibitor	Promega	CAT# N235A
Rotenone	Sigma	CAT# R8875-1G
Galactose Medium	This Paper	N/A
10 mM D-(+)-galactose in Glucose-free DMEM without phenol red	This Paper	N/A
D-(+)-galactose	Sigma	CAT# G5388-100G
Glucose-free DMEM without phenol red	Gibco	CAT# A14430-01
Dasatinib	LC laboratories	CAT# D-3307
6j	Wang et al., 2016	N/A
Ponatinib	Selleckchem	CAT# S1490
Necrostatin-1	Biovision	CAT# 1864-5
KW 2449	Selleckchem	CAT# S2158
APY-69	Astatech	CAT# 77049
GNF-2	Selleckchem	CAT# S2899
Imatinib Mesylate	Selleckchem	CAT# STI571
Omipalisib	Selleckchem	CAT# S2658
Taselisib	Selleckchem	CAT# 7103
PI-103	Selleckchem	CAT# S1038
Apitolosib	Selleckchem	CAT# S2696
Bosutinib	Selleckchem	CAT# S1014
Saracatinib	Selleckchem	CAT# S1006
Foretinib	Med Chem Express	CAT# HY-10338
Ibrutinib	Selleckchem	CAT# S2680
LIMKi3	Ross-Macdonald, et al., 2008	N/A
TH251	Kashima et al., 2017	N/A
Crizotinib	Selleckchem	CAT# S1068
Staurosporine	LC Laboratories	CAT# S-9300
rATP	Promega	CAT# E601B
Critical Commercial Assays		
Cell Titer-Glo 2.0	Promega	CAT# G294A
CellTox-Green	Promega	CAT# G8741

(*Continued on next page*)

Continued		
REAGENT or RESOURCE	SOURCE	IDENTIFIER
Experimental Models: Cell Lines		
HEK-293 Cells	ATCC	CAT# CRL-1573
HeLa Cells	ATCC	CAT# CCL-2
Oligonucleotides		
Cas9 guide sequence crRNA: GGCTATCAGAGTCGAAGATG	IDT – This Paper	N/A
Primers to the CDK2 locus were directed to 540 bp upstream of the CDK2 stop codon: GACCAGCTCTTCCGGATCTTTCGGA	IDT – This Paper	N/A
Primers to the CDK2 locus were directed to 567 bp downstream of the CDK2 stop codon: GCTGAGTCTTCAGTCTCCCAGCCT	IDT – This Paper	N/A
Recombinant DNA		
pFN31K	Promega	CAT# N131A
pFC32K	Promega	CAT# N134A
Transfection Carrier DNA	Promega	CAT# E488A
Software and Algorithms		
Graphpad Prism 7.03	Graphpad	RRID: SCR_002798; https://www.graphpad.com/scientific-software/prism/
KinMap (beta) Kinase Dendrogram Creator	KinHub	http://kinhub.org/kinmap/index.html#
Other		
4D-Nucleofector System	Lonza	http://www.lonza.com/products-services/bio-research/transfection/nucleofector-devices/4d-nucleofector-system.aspx
ImageQuant LAS 4010 gel imager	GE	http://www.gelifesciences.com/webapp/wcs/stores/servlet/catalog/en/GELifeSciences-us/products/AlternativeProductStructure_16010/28955811
SeeBlue Plus2 molecular weight marker	Invitrogen	CAT# LC5925
LV200 Bioluminescence imaging system	Olympus	https://www.olympus-lifescience.com/en/microscopes/inverted/lv200/?apd=1&adid=lv200&acid=enbgle&gclid=EAIaIQobChMIwvH2luL61glVBqlpCh0VrgiiEAAYASAAEgJGrvD_BwE#!cms[tab]=%2Fmicroscopes%2Finverted%2Flv200%2Ffeatures
ImagEM X2 EM-CCD camera	Hamamatsu	https://www.hamamatsu.com/us/en/C9100-23B.html
Nunc Lab-Tek II 8-well chambered coverslips	Thermo Fisher	CAT# 155409
AVANCE III HD 400 MHz spectrometer1H-NMR 400 MHz spectrometer	Bruker	https://www.bruker.com/products/mr/nmr/avance-iii-hd/overview.htmlAvance
Kinetix 5 µm EVO C18 100 Å Analytical HPLC column (2.1 x 30 mm)	Phenomenex	CAT# 00A-4633-AN
Xbridge C18 3.5 µm Analytical HPLC column (4.6 x 50 mm)	Waters	CAT# 186003031
LC-MS: Alliance HPLC w/ 3100 SQ Detector 2	Waters	http://www.waters.com/waters/en_US/Alliance-HPLC-System/nav.htm?cid=534293&locale=en_USAlliance HPLC w/ 3100 SQ Detector 2
LC Prep 150 Reverse-phase prep HPLC	Waters	http://www.waters.com/waters/en_US/Prep-150-LC-System/nav.htm?cid=134727002&locale=en_USLC Prep 150
Xbridge Prep C18 5 µm OBD 30 x 250 mm column prep HPLC	Waters	CAT# 186004025
TripleTOF 5600+ High resolution mass spectrometer	CIEX	https://sciex.com/products/mass-spectrometers/qtof-systems/tripletof-systems/tripletof-5600-system
GloMax Discover Luminometer	Promega	CAT# GM3000

CONTACT FOR REAGENT AND RESOURCE SHARING

Further information and requests for resources and reagents should be directed to and will be fulfilled by the Lead Contact Matthew B. Robers. Matt.Robers@promega.com

EXPERIMENTAL MODEL AND SUBJECT DETAILS

HEK-293 cells (ATCC) or HeLa cells (ATCC) were cultured in DMEM (Gibco) + 10% FBS (GE Healthcare), with incubation in a humidified, 37°C/5% CO_2 incubator.

METHOD DETAILS

Cell Transfections and BRET Measurements

N- or C-terminal NanoLuc/Kinase fusions were encoded in pFN31K or pFC32K expression vectors (Promega), including flexible Gly-Ser-Ser-Gly linkers between Nluc and each full-length kinase. Optimal orientations for each construct are described in Table S1. For cellular BRET target engagement experiments, HEK-293 or HeLa cells were transfected with NLuc/target fusion constructs using FuGENE HD (Promega) according to the manufacturer's protocol. Briefly, Nluc/target fusion constructs were diluted into Transfection Carrier DNA (Promega) at a mass ratio of 1:10 (mass/mass), after which FuGENE HD was added at a ratio of 1:3 (μg DNA: μL FuGENE HD). 1 part (vol) of FuGENE HD complexes thus formed were combined with 20 parts (vol) of HEK-293 cells suspended at a density of 2 x 10^5 per mL, followed by incubation in a humidified, 37°C/5% CO_2 incubator for 20 hr. For broad kinase profiling experiments, kinase transfections were performed in 96-well plates using plasmid DNAs arrayed based on energy transfer probe affinity. To simplify the work flow, energy transfer probes were binned based on their optimal concentrations for each kinase target. Based on these groupings, the work flow could be simplified and 178 individual kinases could be queried by a single person, in a single day. The analysis can be performed without any automated liquid handling instruments or robotics. Following transfection, cells were washed and resuspended in Opti-MEM. BRET assays were performed in white, 96-well plates (Corning) at a density of 2 x 10^4 cells/well. All chemical inhibitors were prepared as concentrated stock solutions in DMSO (Sigma-Aldrich) and diluted in Opti-MEM (unless otherwise noted) to prepare working stocks. Cells were equilibrated for 2 hr with energy transfer probes and test compound prior to BRET measurements. Energy transfer probes were prepared at a working concentration of 20X in tracer dilution buffer (12.5 mM HEPES, 31.25% PEG-400, pH 7.5). For target engagement analysis, the energy transfer probes were added to the cells at concentrations optimized for each target, as described in Table S1. For analysis of DDR1 and DDR2 with compound 6j, energy transfer probe 6 was used at a concentration of 300 nM and 330 nM, respectively. To measure BRET, NanoBRET NanoGlo Substrate and Extracellular NanoLuc Inhibitor (Promega) were added according to the manufacturer's recommended protocol, and filtered luminescence was measured on a GloMax Discover luminometer equipped with 450 nm BP filter (donor) and 600 nm LP filter (acceptor), using 0.5 s integration time. Milli-BRET units (mBU) are calculated by multiplying the raw BRET values by 1000. Apparent tracer affinity values (EC_{50}) were determined using the sigmoidal dose-response (variable slope) equation available in GraphPad Prism (Equation 1);

$$Y = Bottom + (Top\text{-}Bottom)/(1 + 10\char`^((LogEC50\text{-}X)*HillSlope)). \qquad \text{(Equation 1)}$$

Competitive displacement data were then plotted with GraphPad Prism software and data were fit to Equation 1 to determine the IC_{50} value.

For fractional occupancy determination in kinase profiling experiments, the following equation (Equation 2) was used;

$$\%\ Occupancy = [1 - (X - Z)/(Y - Z)]*100 \qquad \text{(Equation 2)}$$

where X = BRET in the presence of the test compound and energy transfer probe, Y = BRET in the presence of only energy transfer probe, and Z = BRET in the absence of the energy transfer probe and test compound. Predicted biochemical occupancy at the chosen drug dose for kinase profiling experiments was determined from the K_d value reported previously (Davis et al., 2011) using a variation of the Langmuir Isotherm (Hulme and Trevethick, 2010) (Equation 3);

$$\%\ Occupancy = [[Drug]/(K_d + [Drug])]*100 \qquad \text{(Equation 3)}$$

For all BRET data shown, no individual data points were omitted.

Cellular ATP Depletion

In order to evaluate the effect of endogenous ATP on target engagement parameters, cells were depleted of ATP by treatment with rotenone, a known inhibitor of mitochondrial ATP synthesis that blocks complex I. This method was adapted from previous reports (Marroquin et al., 2007; Swiss et al., 2013) that use a glucose-free galactose medium to sensitize cultured mammalian cells to mitochondrial toxicants, and adjusted to fit the BRET based target engagement workflow described above. Briefly, HEK-293 cells

expressing the kinase/NLuc fusion of interest were prepared by transient transfection as described in the Cell transfection, treatments, and BRET measurements section. The cells were harvested by trypsinization, pelleted by centrifugation, and resuspended in galactose medium, which consisted of 1 mM HEPES (Gibco), 2 mM L-glutamine (Gibco), 1 mM sodium pyruvate (Gibco), and 10 mM D-(+)-galactose (Sigma) in glucose-free DMEM without phenol red (Gibco A14430-01). The cell density was adjusted to 2 x 10^5 cells/mL, after which 1000X rotenone (1 mM in DMSO) was added to achieve a final concentration of 1 μM in the cell suspension. Subsequently, the cells were used in target engagement workflows as described above in the cell transfection, treatments, and BRET measurements section, except that working stocks of all chemical inhibitors, NanoGlo Substrate, and Extracellular NanoLuc Inhibitor were prepared in galactose medium rather than OptiMEM. Relative ATP levels and membrane integrity of the cell suspension were monitored using a multiplex of the Cell Titer-Glo 2.0 Assay (Promega) and the CellTox Green assay (Promega) performed as described in the manufacturer's protocols. After a 2 hour treatment, this protocol routinely reduces the total ATP in the cell suspension by 90−95% relative to those maintained in glucose rich media, and without loss of membrane integrity (Figure S5). For assays in permeabilized format, cells were permeabilized by treatment with 50 μg/mL digitonin, and Extracellular NanoLuc Inhibitor was omitted from the NanoGlo Substrate solution.

Endogenous Tagging Using CRISPR/Cas9

To assess the target engagement behavior of CDK2-NanoLuc fusion expressed at endogenous levels, NanoLuc was introduced as a fusion by homologous directed repair of a CRISPR-Cas9 double-strand break at the CDK2 locus in HEK293 cells. The genome edit was designed to insert the NanoLuc coding sequence and thereby create a fusion of NanoLuc to the C-terminus of the CDK2 protein. The Cas9 guide sequence crRNA was directed to the sequence GGCTATCAGAGTCGAAGATG (the PAM site is GGG) and generates a double strand break 9 bp upstream of the stop codon for CDK2. The donor DNA sequence for homologous directed repair was contained in a circular double-stranded plasmid DNA, and included 500bp of genomic CDK2 DNA sequence upstream of the stop codon, the NanoLuc coding sequence including a four amino acid flexible peptide linker (GSSG), and 450bp of CDK2 3'UTR genomic sequence. Cas9 protein (Aldevron His-sNLS-Myc-spCas9) was incubated with tracrRNA:crRNA (IDT) at equimolar ratios to create a ribonucleoprotein (RNP) complex. The RNP and donor DNA plasmid was introduced to 2x10^5 HEK293 cells via electroporation using a Lonza 4D NucleoFector. After 6 days, the electroporated cells were diluted and plated in 96 well plates to isolate single colonies. Single colony isolates showing light production three-fold over background were collected and tested for stable growth and light generation, and a small number of clonal cell lines were screened by PCR for NanoLuc insertion using CDK2 genomic primers that were outside the homology regions used in the donor DNA plasmid. One clonal cell line, B10, was isolated that was homozygous for insertion, Figure 1A. The PCR amplicon from the homozygous insertion CDK2-NanoLuc cell line was remade with the high fidelity DNA polymerase Q5 (New England Biolabs) and ligated into a bacterial plasmid for DNA sequencing. 24 plasmid clones were sequenced, and all 24 showed perfect homology throughout the CDK2 regions upstream and downstream of the NanoLuc insertion point. Protein was isolated from the homozygous insertion CDK2-NanoLuc cell line to perform a Western using anti-CDK2 (Figure S2). The antibody stained blot showed that CDK2-staining band now ran at the expected higher molecular weight of the CDK2-NanoLuc fusion. PCR Characterization; Primers to the CDK2 locus were directed to 540 bp upstream of the CDK2 stop codon (GACCAGCTCTTCCGGATCTTTCGGA) and 567 bp downstream of the CDK2 stop codon (GCTGAGTCTT CAGTCTCCCAGCCT). In HEK-293, the resulting amplicon is 1,110 bp; a successful insertion of the NanoLuc coding region, 534bp, would result in an amplicon size of 1,644 bp. Clonal line B10 shows only the 1,644 bp amplicon, and represents homozygous insertion of NanoLuc with no "wild-type" amplicon. Clonal line C5 is included to illustrate a heterozygous insertion of NanoLuc with both amplicons present. The PCR conditions used 100ng of genomic DNA combined with 20 pmol of each primer and GoTaq® Hot Start Green Master Mix (Promega) in a total volume of 50μl. 30 cycles of PCR were performed with an annealing temperature of 61°C for 20 seconds and an extension temperature of 72°C for 1 minute. 5 μl of the reactions were run on a 1.2% agarose gel stained with Ethidium Bromide; image capture was done with a LAS4010 (GE). Western Blots were performed to assess the molecular weight of the CDK2-NanoLuc fusion protein in the clonal cell lines. The expected molecular weight of CDK2 is 34KDa and the CDK-NanoLuc fusion is 53KDa. Cells from the clonal lines B10 and C5 were harvested by trypsinization, the cell density determined, and 1−2 x 10^6 cells were pelleted, washed with PBS, pelleted again and the cell pellets were frozen at −80°C for storage. Samples were thawed and re-suspended in PBS containing 0.1% BSA, then added to an equal volume of Loading Buffer (Bio-RAD). Samples were separated by SDS/PAGE (BIO-RAD) according to the manufacturer's recommendations and included the SeeBlue Plus2 molecular weight marker (Invitrogen). Western blots were performed using the SuperSignal West Pico Detection Kit (Thermo) following the manufacturers recommendations. Briefly, after electrophoresis, proteins were transferred to a PVDF membrane and blocked overnight at 4°C. Blots were incubated in primary antibody (Cell Signaling Technologies #25465) for one hour at room temperature on a rocking platform, then washed six times. Blots were similarly incubated in secondary antibody (HRP-conjugate, 1:1,000 dilution), washed six times and briefly exposed to ECL substrate. Image capture was done with a LAS4010 (GE). The loading control for the CDK2 Western is shown immediately below and followed the same protocol as above, but the primary antibody was against p38 (Abcam ab170099).

Bioluminescence Imaging

All imaging experiments were performed using the LV200 bioluminescence imaging system (Olympus) equipped with an ImagEM X2 EM-CCD camera (Hamamatsu) and a 60x, 1.4 NA objective. HEK293 cells transiently transfected with the indicated expression construct (1:10 dilution in carrier DNA) were plated onto Nunc Lab-Tek II 8-well chambered coverslips (Thermo Fisher) in 400 μl growth medium (DMEM + 10% FBS) at a density of 8X10^5 cells per well. After 24 h of incubation at 37 °C, 100 μl of Nano-Glo

Live Cell Reagent (Promega) was added. All images were acquired with cellSens software (Olympus) using electron multiplying (EM) gain of 600 and an exposure time of 3 seconds. Each image was generated using a average projection of 10 images. Generation of average projections, linear adjustments of dynamic range, and pseudocolor rendering were performed using Image J image processing software (Fiji package). Scale bar = 20 μm.

Chemical Synthesis

General Information

All solvents were purchased from Sigma or Fisher Scientific and used without purification. NanoBRET 590 SE was obtained from Promega Corp. Madison, WI. ^{1}H-NMR spectra were recorded on a Bruker Avance 400 MHz spectrometer or a Bruker Ascend 400 MHz spectrometer. Chemical shifts (δ) are quoted in parts per million (ppm) and referenced to the residual solvent peak. Multiplicities are denoted as s-singlet, d-doublet, t-triplet, q-quartet and quin-quintet and derivatives thereof (br denotes a broad resonance peak). Coupling constants are given in Hz and round to the nearest 0.1 Hz. Mass spectra were recorded on a Waters SQ Detector 2 (LC-MS) and purity (≥95 %) determined by reverse-phase high pressure liquid chromatography (RP-HPLC) using a Kinetex 5 μm EVO C18 100 Å LC Column 30 × 2.1 mm column or a Phenomenex Synergi 2.5 μm Max-RP 100 Å LC column. High resolution mass spectrum (HRMS) were recorded on a SCIEX Triple TOF 5600 spectrometer. Compounds were purified on a Waters LC Prep 150 using a Waters XBridge Prep C18 OBD 30x250mm column. Standard Method 1: Initial−90% aqueous (0.1% TFA in H_2O), 10% acetonitrile to 0% aqueous, 100% acetonitrile, 30 min linear gradient.

Chemical Synthesis of Energy Transfer Probe 1

2-(4-((4-((4-aminobutyl)amino)-6-((5-cyclopropyl-1H-pyrazol-3-yl)amino)pyrimidin-2-yl)amino)phenyl)acetonitrile

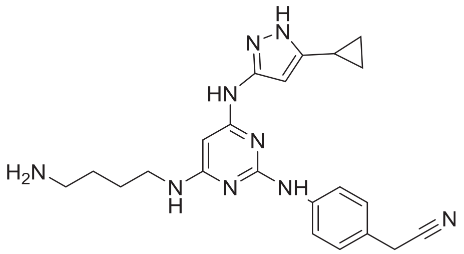

To a 5 mL pressure tube was added 1,4-diaminobutane (0.9 g, 10.3 mmol) followed by 2-(4-((4-chloro-6-((5-cyclopropyl-1H-pyrazol-3-yl)amino)pyrimidin-2-yl)amino)phenyl)acetonitrile (Statsuk et al., 2008) (50.0 mg, 0.14 mmol). The tube was capped and the mixture was heated to 120 °C overnight. The tube was cooled to ambient temperature, uncapped and was diluted with 50 mL of ethyl acetate, giving a yellow precipitate. The crude mixture was transferred to a 250 mL separatory funnel and was washed with a saturated aqueous solution of ammonium chloride (25 mL). The organic phase was retained, washed with 25 mL of water then 25 mL of brine solution. The organic phase was dried over Na_2SO_4, filtered and concentrated to give a yellow-white solid. The solid was dissolved in water (8.0 mL) and was subjected to RP-HPLC preparative purification using Standard Method 1. Product containing fractions were analyzed by HPLC, pooled and were concentrated to afford the product (22.7 mg, 39.8%) as a yellow solid. ^{1}H NMR (400 MHz, methanol-*d*) δ 7.90 (s, 1H), 7.63 (d, *J* = 8.3 Hz, 2H), 7.41 (d, *J* = 8.3 Hz, 2H), 5.60 (s, 1H), 3.91 (s, 2H), 3.45 (s, 2H), 2.98 (m, 2H), 1.93 (m, 1H), 1.73 (m, 4H), 1.02 (m, 2H), 0.72 (m, 2H). HRMS (ESI) calcd for $C_{22}H_{28}N_9$ $[M+H]^+$: 418.2468. Found: 418.2452 .

2-(4-((4-((4-aminobutyl)amino)-6-((5-cyclopropyl-1H-pyrazol-3-yl)amino)pyrimidin-2-yl)amino)phenyl)acetonitrile-NanoBRET 590(Energy Transfer Probe 1)

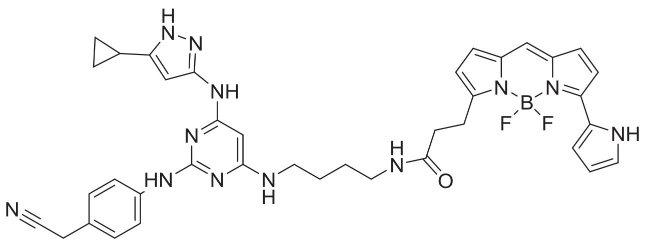

To a stirred solution of 2-(4-((4-((4-aminobutyl)amino)-6-((5-cyclopropyl-1H-pyrazol-3-yl)amino)pyrimidin-2-yl)amino)phenyl) acetonitrile (3.9 mg, 0.009 mmol) in anhydrous DMF (1.0 mL) was added *N*-*N*-diisopropylethylamine (8.4 μL, 0.05 mmol). The mixture was stirred for 10 min and NanoBRET 590 SE (4.4 mg, 0.01 mmol) was added. The reaction mixture was capped and stirred in the dark for 3 hrs. The reaction was quenched with 6 mL of a solution of acetonitrile, water, TFA (1:1:0.01) and was subjected to RP-HPLC preparative purification using Standard Method 1. Product containing fractions were analyzed by HPLC for purity, pooled and were concentrated under reduced pressure affording compound 1 (2.8 mg, 40.9%) as a blue film. ^{1}H NMR (400 MHz, methanol-*d*) δ 7.58

(m, 2H), 7.37 (d, J = 8.3 Hz, 2H), 7.16 (m, 4H), 6.97 (d, J = 4.6 Hz, 1H), 6.87 (m, 1H), 6.34 (m, 2H), 6.30 (d, J = 4.0 Hz, 1H) 3.41 (s, 2H), 2.62 (t, J = 7.5 Hz, 1H) 1.91 (m, 1H), 1.59 (s, 4H), 1.29 (s, 1H), 1.02 (m, 2H), 0.90 (m, 2H), 0.72 (m, 2H). HRMS (ESI) calcd for $C_{38}H_{40}BF_2N_{12}O$ $[M+H]^+$: 729.3509. Found: 729.3512.

Chemical Synthesis of Energy Transfer Probe 2

tert-butyl (18-(4-((6-(2,6-dichlorophenyl)-8-methyl-7-oxo-7,8-dihydropyrido[2,3-d]pyrimidin-2-yl)amino)phenoxy)-15-oxo-3,6,9,12-tetraoxa-16-azaoctadecyl)carbamate

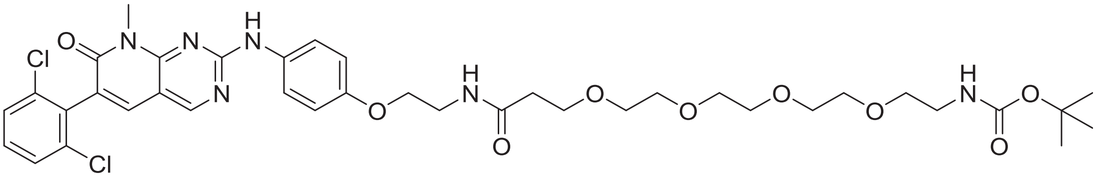

To a 5 mL amber vial was added 2,5-dioxopyrrolidin-1-yl 2,2-dimethyl-4-oxo-3,8,11,14,17-pentaoxa-5-azaicosan-20-oate (10.0 mg, 0.022 mmol), 2-((4-(2-aminoethoxy)phenyl)amino)-6-(2,6-dichlorophenyl)-8-methylpyrido[2,3-d]pyrimidin-7(8H)-one (Klutchko et al., 1998) (4.7 mg, 0.01 mmol) and DMF (0.5 mL). To the stirred solution was added *N*-*N*-diisopropylethylamine (9.0 μL, 0.05 mmol) and the mixture was allowed to stir for 2 hrs. The reaction mixture was diluted with a solution of acetonitrile, water, TFA (1:1:0.01) to 6 mL and was subjected to RP-HPLC preparative purification using Standard Method 1. Product containing fractions were analyzed by HPLC for purity, pooled and concentrated to afford the product (5.2 mg, 62.8%) as a yellow solid. HRMS (ESI) calcd for $C_{38}H_{49}Cl_2N_6O_9$ $[M+H]^+$: 803.2938. Found: 803.2929.

1-amino-N-(2-(4-((6-(2,6-dichlorophenyl)-8-methyl-7-oxo-7,8-dihydropyrido[2,3-d]pyrimidin-2-yl)amino)phenoxy)ethyl)-3,6,9,12-tetraoxapentadecan-15-amide, TFA salt

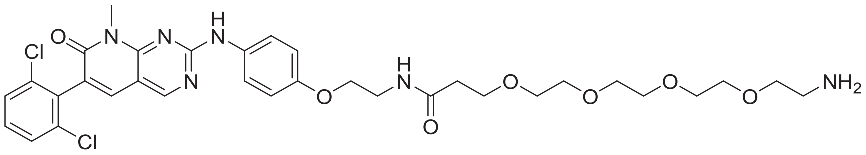

tert-butyl (18-(4-((6-(2,6-dichlorophenyl)-8-methyl-7-oxo-7,8-dihydropyrido[2,3-d]pyrimidin-2-yl)amino)phenoxy)-15-oxo-3,6,9,12-tetraoxa-16-azaoctadecyl)carbamate was taken up in DCM (1.0 mL) and diisopropylsilane (100 μL) was added. To the stirred solution was added trifluoroacetic acid (1.0 mL) and the reaction was allowed to stir for 90 min. Volatiles were removed under reduced pressure giving a colorless oil that was treated with diethyl ether (10 mL) affording a white solid. The ether was decanted and the resulting solid was dried under high vacuum for 10 min. An additional 10 mL portion of diethyl ether was added to the reaction residue, decanted and the resulting solid was dried under high vacuum for 1 hr.

1-amino-N-(2-(4-((6-(2,6-dichlorophenyl)-8-methyl-7-oxo-7,8-dihydropyrido[2,3-d]pyrimidin-2-yl)amino)phenoxy)ethyl)-3,6,9,12-tetraoxapentadecan-15-amide-NanoBRET 590 (Energy Transfer Probe 2)

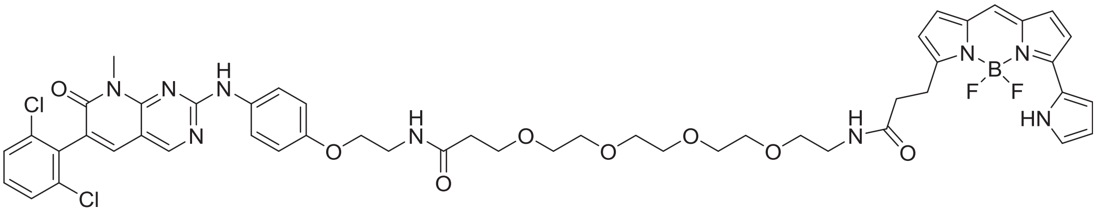

Crude 1-amino-N-(2-(4-((6-(2,6-dichlorophenyl)-8-methyl-7-oxo-7,8-dihydropyrido[2,3-d]pyrimidin-2-yl)amino)phenoxy)ethyl)-3,6,9,12-tetraoxapentadecan-15-amide-TFA salt was taken up in DMF (1.0 mL), was treated with *N*-*N*-diisopropylethylamine (11.3 μL, 0.07 mmol) and the mixture was stirred for 10 min. NanoBRET 590 SE (3.3 mg, 0.008 mmol) was added and the mixture was allowed to stir in the dark for 2 hr. The reaction mixture was diluted with a solution of acetonitrile, water, TFA (1:1:0.01) to 6 mL and was subjected to RP-HPLC prep purification using Standard Method 1. Product containing fractions were analyzed by HPLC for purity, pooled and were concentrated under reduced pressure affording compound 2 (1.2 mg, 18% -two steps) as a blue film. ^1H NMR (400 MHz, methanol-*d*) δ 8.68 (s, 1H), 7.73 (s, 1H), 7.65 (s, 1H), 7.50 (d, J = 0.9 Hz, 1H), 7.48 (s, 1H), 7.40 (m, 1H), 7.19 (m, 1H) 7.18 (s, 1H) 7.15 (s, 1H), 6.95 (m, 3H), 6.88 (d, J = 3.9 Hz, 1H), 6.33 (m, 1H), 6.30 (d, J = 4.0 Hz, 1H), 4.04

(t, J = 10.8 Hz, 2H), 3.69 (m, 2H), 3.55 (m, 17H), 3.61 (m, 3H), 3.26 (m, 2H), 2.63 (t, J = 7.6 Hz, 2H), 2.45 (t, J = 6.0 Hz, 2H). HRMS (ESI) calcd for $C_{49}H_{53}BCl_2F_2N_9O_8$ $[M+H]^+$: 1014.3455. Found: 1014.3461.

Chemical Synthesis of Energy Transfer Probe 3

3-(4-morpholino-6-(piperazin-1-ylmethyl)thieno[3,2-*d*]pyrimidin-2-yl)phenol-NanoBRET 590 (Energy Transfer Probe 3)

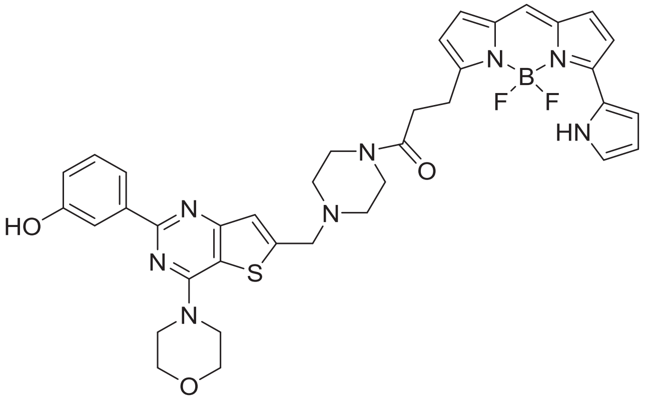

To a stirred solution of 3-(4-morpholino-6-(piperazin-1-ylmethyl)thieno[3,2-*d*]pyrimidin-2-yl)phenol (Nacht et al., 2013) (8.0 mg, 0.019 mmol) in anhydrous DMF (1.0 mL) was added *N*-*N*-diisopropylethylamine (17.0 μL, 0.097 mmol). The mixture was stirred for 10 min, NanoBRET 590 SE (9.1 mg, 0.021 mmol) was added and the reaction mixture was capped and stirred in the dark for 2 hr. The reaction was quenched with 6 mL of a solution of acetonitrile, water, TFA (1:1:0.01) and was subjected to RP-HPLC preparative purification using Standard Method 1. Product containing fractions were analyzed by HPLC for purity, pooled and were concentrated under reduced pressure affording compound 3 (9.9 mg, 70.0%) as a blue film. ^{1}H NMR (400 MHz, methanol-*d*) δ 7.70 (s, 1H) 7.67 (s, 1H), 7.47 (s, 1H), 7.43 (t, J = 7.3 Hz, 1H), 7.25 (s, 1H), 7.21 (m, 2H), 7.18 (m, 1H), 7.10 (m, 1H), 7.02 (d, J = 4.6 Hz, 1H), 6.93 (D, J = 4.0 Hz, 1H), 6.33 (m, 2H), 4.27 (t, J = 4.8 Hz, 4H), 4.08 (s, 2H), 3.92 (t, J = 5.0 Hz, 4H), 3.71 (s, 2H), 3.64 (s, 1H), 2.86 (t, J = 7.8 Hz, 2H), 2.68 (t, J = 5.9 Hz, 4H). HRMS (ESI) calcd for $C_{37}H_{3}8BF_2N_8O_3S$ $[M+H]^+$: 723.2849. Found: 723.2830.

Chemical Synthesis of Energy Transfer Probe 4

N-(3-((4-(3-aminopropyl)piperazin-1-yl)methyl)-2-(trifluoromethyl)phenyl)-3-(imidazo[1,2-*b*]pyridazin-3-ylethynyl)-4-methylbenzamide-NanoBRET 590 (Energy Transfer Probe 4)

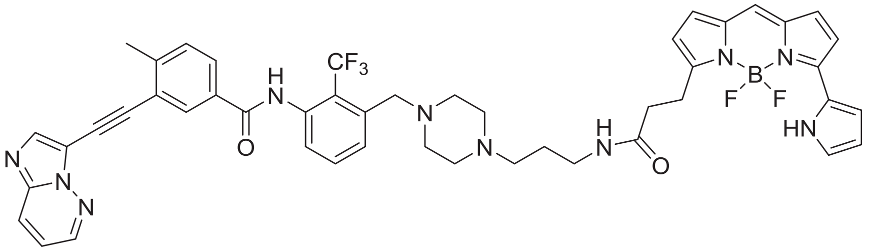

N-*N*-diisopropylethylamine (5.6 μL, 0.032 mmol) was added to a solution of *N*-(4-((4-(3-aminopropyl)piperazin-1-yl)methyl)-3-(trifluoromethyl)phenyl)-3-(imidazo[1,2-b]pyridazin-3-ylethynyl)-4-methylbenzamide (Fauster et al., 2015) (7.5 mg, 0.013 mmol) in anhydrous DMF (1 mL) and the mixture stirred for 10 min. NanoBRET 590 SE (5.2 mg, 0.012 mmol) was added and the reaction stirred in the dark for 4 hrs followed by removal of the solvent under vacuum. The crude product was purified via preparative TLC (5 % methanol in dichloromethane) affording compound 4 (10.7 mg, 93 %) as a purple solid. ^{1}H NMR (400 MHz, $CDCl_3$) δ 10.40 (s, 1H), 8.50 (dd, J = 4.4, 1.6 Hz, 1H), 8.07 (m, 2H), 8.01 (dd, J = 9.2, 1.6 Hz, 1H), 7.93 (br s, 1H), 7.82 (m, 3H), 7.63 (d, J = 9.0 Hz, 1H), 7.42 (d, J = 8.0 Hz, 1H), 7.19 – 7.12 (m, 3H), 7.03 (d, J = 4.6 Hz, 1H), 7.00 – 6.95 (m, 2H), 6.85 (m, 2H), 6.40 – 6.28 (m, 2H), 3.64 (s, 2H), 3.50 (s, 2H), 3.35 (t, J = 7.3 Hz, 4H), 2.43 – 2.36 (m, 9H), 1.62 (p, J = 6.2 Hz, 2H), 1.26 (s, 4H). MS (ESI) calcd for $C_{47}H_{45}BF_5N_{10}O_2$ $[M + H]^+$: 887.37. Found: 887.73.

Chemical Synthesis of Energy Transfer Probe 5

Tert-butyl (15-(4-(4-((5-chloro-4-((2-(methylcarbamoyl)phenyl)amino)pyrimidin-2-yl)amino)phenyl)piperazin-1-yl)-15-oxo-3,6,9,12-tetraoxapentadecyl)carbamate.

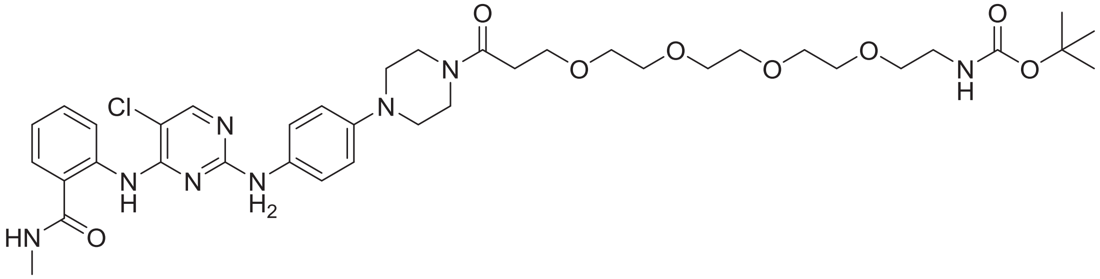

To a round bottom flask was charged 2,2-dimethyl-4-oxo-3,8,11,14,17-pentaoxa-5-azaicosan-20-oic acid (20.0 mg, 0.046 mmol), 2-((5-chloro-2-((4-(piperazin-1-yl)phenyl)amino)pyrimidin-4-yl)amino)-*N*-methylbenzamide (25.0 mg, 0.069 mmol) and HATU (20.8 mg, .058 mmol). The solids were taken up in anhydrous DMF (1.0 mL), *N*,*N*-diisopropylethylamine (23.9 μL, 0.137 mmol) was added and the mixture was allowed to stir for 3 hr. Volatiles were removed under reduced pressure and the resulting residue was dried under high vacuum for 2 hr. The crude was subjected to flash chromatography (0% to 10% methanol/DCM gradient). Product containing fractions were pooled and concentrated affording the product (25.0 mg, 55.8%) as a white solid. ^{1}H NMR (400 MHz, $CDCl_3$) δ 11.19 (s, 1H), 8.63 (d, *J* = 8.4 Hz, 1H), 8.00 (s, 1H), 7.68 (m, 1H), 7.50 (d, *J* = 1.6 Hz, 1H), 7.44 (d, *J* = 8.6 Hz, 2H), 7.37 (t, *J* = 7.9 Hz, 1H), 7.06 (t, *J* = 7.5 Hz, 1H), 6.88 (d, *J* = 8.7 Hz, 2H), 6.46 (m, 1H), 5.15 (s, 1H), 3.79 (m, 6H), 3.59 (m, 2H), 3.50 (m, 4H) 3.28 (m, 4H), 3.09 (m, 4H), 3.00 (d, *J* = 4.8 Hz, 2H), 2.64 (m, 3H), 1.56 (d, *J* = 6.7 Hz, 2H), 1.43 (s, 15H) 1.05 (m, 2H). MS (ESI) calcd for $C_{38}H_{54}ClN_8O_8$ [M + H]$^+$: 785.38. Found: 785.60.

2-((2-((4-(4-(1-amino-3,6,9,12-tetraoxapentadecan-15-oyl)piperazin-1-yl)phenyl)amino)-5-chloropyrimidin-4-yl)amino)-N-methylbenzamide-TFA salt

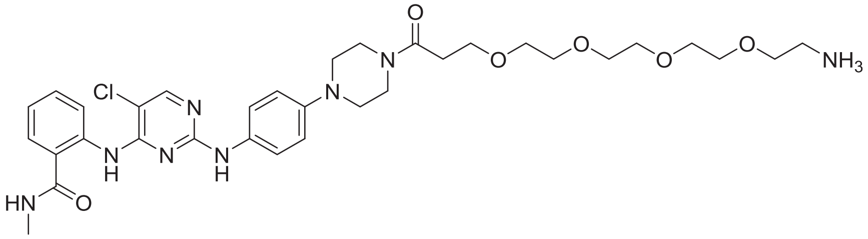

Tert-butyl (15-(4-(4-((5-chloro-4-((2-(methylcarbamoyl)phenyl)amino)pyrimidin-2-yl)amino)phenyl)piperazin-1-yl)-15-oxo-3,6,9,12-tetraoxapentadecyl)carbamate (25.0 mg, 0.032 mmol) was dissolved in 2.5 mL of DCM and triisopropylsilane (250 μL, 0.031 mmol) and TFA (2.5 mL) was added. The mixture was capped and stirred for 3 hr. Volatiles were removed under reduced pressure giving a yellow oil and 5 mL of diethyl ether was added giving a white precipitate. The ether was decanted and an additional 5 mL portion was added. The suspension was sonicated and the ether was decanted. The resulting residue was dried under high vacuum overnight affording the product (18.9 mg, 77.9%) as a white solid. MS (ESI) calcd for $C_{33}H_{46}ClN_8O_6$ [M + H]$^+$: 785.23. Found: 785.32.

2-((2-((4-(4-(1-amino-3,6,9,12-tetraoxapentadecan-15-oyl)piperazin-1-yl)phenyl)amino)-5-chloropyrimidin-4-yl)amino)-N-methylbenzamide-NanoBRET 590 (Energy Transfer Probe 5)

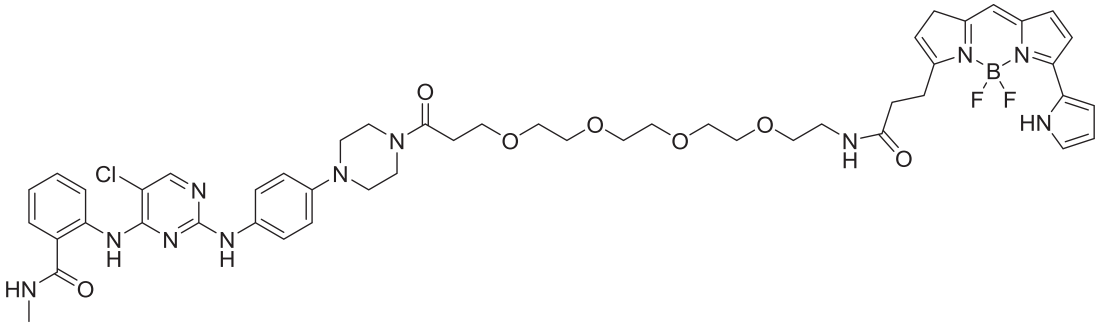

Primary amine 2-((2-((4-(4-(1-amino-3,6,9,12-tetraoxapentadecan-15-oyl)piperazin-1-yl)phenyl)amino)-5-chloropyrimidin-4-yl)amino)-N-methylbenzamide (55.0 mg, 0.08 mmol) was charged into a round bottom flask and was taken up in anhydrous DMF (5.0 mL). To the stirred solution was added *N*,*N*-diisopropylethylamine (69.9 uL, 0.40 mmol) then NaoBRET 590 SE (41.1 mg, 0.096 mmol). The mixture was capped and stirred for 2 hr, judged complete by the disappearance of the starting amine by HPLC. The reaction was quenched with 6 mL of a solution of acetonitrile, water, TFA (1:1:0.01) and was subjected to RP-HPLC prep

purification using Standard Method 1. Product containing fractions were analyzed by HPLC for purity, pooled and were concentrated under reduced pressure affording the product 5 (64.8 mg, 81.0%) as a blue film. ^{1}H NMR (400 MHz, DMSO-*d*) δ 11.60 (s, 1H), 11.40 (s, 1H), 9.24 (s, 1H), 8.73 (m, 2H), 8.16 (s, 1H), 8.00 (t, *J* = 5.6 Hz, 1H), 7.74 (dd, *J* = 1.6 Hz, *J* = 7.9 Hz, 1H), 7.48 (m, 4H), 7.43 (s, 1H), 7.36 (m, 1H), 7.32 (d, *J* = 4.6 Hz, 1H), 7.16 (d, *J* = 4.6 Hz, 1H), 7.12 (m, 1H), 7.01 (d, *J* = 4.0 Hz, 1H), 6.90 (d, *J* = 9.0 Hz, 2H), 6.33 (m, 2H), 3.61 (m, 6H), 3.51 (m, 2H), 3.41 (t, *J* = 5.8 Hz, 2H), 3.21 (m, 2H), 3.13 (m, 2H), 3.06 (m, 2H), 3.01 (m, 2H), 2.81 (d, *J* = 4.4 Hz, 2H), 2.61 (t, *J* = 6.6 Hz, 2H). MS (ESI): calcd for $C_{49}H_{58}BClF_2N_{11}O_7$ $[M]^+$ 996.43. Found: 996.55.

Chemical Synthesis of Energy Transfer Probe 6

Tert-butyl (2-(2-hydroxyethoxy)ethyl)carbamate

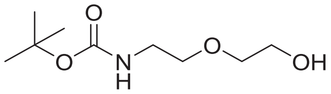

To a stirred solution of 2-(2-Aminoethoxy)ethanol (27.5 g, 261.6 mmol) in EtOAc (100 mL), cooled to 0°C, was added a mixture of Di-*tert*-butyl dicarbonate (60.0 g, 274.6 mmol) in EtOAc (100 mL) dropwise over 1 hr. The mix warmed to rt and was left stirred for 3.5 hr. The crude mixture was washed with 3 × 200 mL portions of water then portion of brine (100 mL). The organic phase was dried over Na_2SO_4 and volatiles were removed under reduced pressure to provide 52.0 g of the Boc-amine (52.01 g, 96.9%) as a colorless oil. ^{1}H NMR (300 MHz, $CDCl_3$) δ 3.74 (t, J = 3.0 Hz, 2H), 3.56 (m, 4H), 3.31 (m, 2H), 1.45 (s, 9H).

Tert-butyl (2-(2-bromoethoxy)ethyl)carbamate

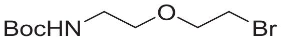

To a stirred solution of t*ert*-butyl (2-(2-hydroxyethoxy)ethyl)carbamate (10.0 g, 48.7 mmol) in THF (100 mL) was added tertabromomethane (24.24 g, 73.1 mmol). The resulting solution was cooled to 0°C and triphenylphosphine (19.17 g, 73.1 mmol) was added in 3 x 5 g portions at 0 min, 15 min and 30 min. At 45 min, a 4.17 g was added. The reaction was allowed to warm to rt and left stirred for 6 hr. The crude reaction mixture was filtered and the filtrate was concentrated to a yellow solid. The solid was dissolved in DCM, absorbed on celite and was dried. The compound was subjected to flash chromatography (0 to 5% MeOH/DCM) affording the product (8.76 g, 67.1%) as a yellow oil. ^{1}H NMR (400 MHz, $CDCl_3$) δ 3.78 (t, J = 6.0 Hz, 2H), 3.56 (t, J = 5.12 Hz, 2H), 3.47 (t, J = 6.0 Hz, 2H) 3.33 (m, 2H), 1.45 (s, 9H).

Tert-butyl (2-(2-(4-(2,2,2-trifluoroacetyl)piperazin-1-yl)ethoxy)ethyl)carbamate

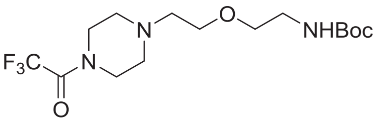

To a solution of *tert*-butyl (2-(2-bromoethoxy)ethyl)carbamate (8.83 g, 32.9 mmol) and trifluoroacetylpiperazine (5.0 g, 27.5 mmol) in ACN (50 mL) was added cesium carbonate (10.7 g, 32.9 mmol) and KI (227.8 g, 1.4 mmol). The mixture was heated to 60°C with stirring for 4 hr. Volatiles were removed under reduced pressure and the resulting residue was dried under hi-vacuum. To the white residue was added EtOAc (100 mL) giving a white suspension that was filtered. The filtrate was washed with two portions of water (50 mL), dried over Na_2SO_4 and was concentrated to a yellow oil that was diluted with DCM, absorbed on celite and was subjected to flash chromatography (0 to 5% MeOH/DCM) affording the product (8.15 g, 80.4%) as a colorless oil. ^{1}H NMR (400 MHz, $CDCl_3$) δ 3.67 (t, J= 5.2, 2H), 3.59 (t, J = 5.0 Hz, 2H), 3.54 (t, J = 5.4 Hz, 2H), 3.47 (t, J = 5.2 Hz, 2H), 3.25 (m, 4H), 2.55 (t, J = 5.4 Hz, 2H), 2.51 (m, 4H), 1.39 (s, 9H).

Tert-butyl (2-(2-(piperazin-1-yl)ethoxy)ethyl)carbamate

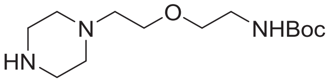

To a stirred solution of *tert*-butyl (2-(2-(4-(2,2,2-trifluoroacetyl)piperazin-1-yl)ethoxy)ethyl)carbamate (5.0 g, 13.5 mmol) in methanol (30 mL) and water (2 mL) was added potassium carbonate (9.35 g, 67.7 mmol). The mixture was stirred for 2 hr then concentrated to dryness. To the residue was added EtOAC (100 mL) giving a white suspension that was filtered then concentrated to ∼10 mL, absorbed on celite and dried. The crude was subjected to flash chromatography (0 to 5% MeOH/DCM) affording the product (2.6 g,

70.0%) as a colorless oil. ^{1}H NMR (400 MHz, $CDCl_3$) δ 3.59 (t, J = 5.6 Hz, 2H), 3.51 (t, J = 5.2 Hz, 2H) 3.30 (m, 2H), 2.92 (t, J = 4.9 Hz, 4H), 2.56 (t, J = 5.7Hz, 2H), 2.48 (m, 4H), 1.44 (s, 9H).

Tert-butyl (2-(2-(4-(6-((5-((2-chloro-6-methylphenyl)carbamoyl)thiazol-2-yl)amino)-2-methylpyrimidin-4-yl)piperazin-1-yl)ethoxy) ethyl)carbamate

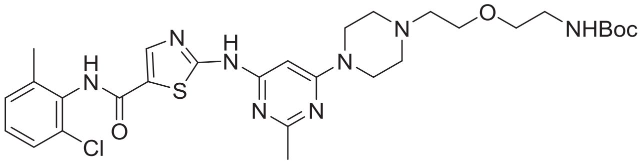

Tert-butyl (2-(2-(piperazin-1-yl)ethoxy)ethyl)carbamate (2.9 g, 10.7 mmol) and N-(2-Chloro-6-methylphenyl)-2-[(6-chloro-2-methyl-4-pyrimidinyl)amino]-5-thiazolecarboxamide (1.1 g, 2.7 mmol) were charged into a round bottom flask. To the solids was added 2-propanol (5 mL), ethylene glycol (5 mL) and diisopropylethylamine (1.9 mL, 10.7 mmol). The mixture was heated to 110°C overnight giving a yellow solid after cooling. To the solid was added 50 mL of water giving a yellow solid that was collected by vacuum filtration and dried under high vacuum overnight. The crude residue was taken up in DCM/methanol, absorbed on celite and was dried to a free-flowing solid. The mixture was subjected to flash chromatography (0 to 5% MeOH/DCM) affording the product (1.08 g, 64.3%) as a white solid. ^{1}H NMR (400 MHz, $CDCl_3$) δ 9.91(s, 1H), 9.85 (s, 1H), 8.24 (s, 1H), 7.41 (d, *J* = 1.9 Hz, 1H), 7.29 (m, 2H), 6.88 (t, *J* = 7.6 Hz, 1H), 6.15 (s, 1H), 4.35 (d, *J* = 9.4 Hz, 9.4 Hz, 2H), 3.74 (d, *J* = 4.7 Hz, 2H), 3.61 (m, 2H), 3.47 (t, *J* = 5.8 Hz, 2H) 3.37 (m, 2H), 3.28 (m, 2H), 3.14 (m, 4H), 2.45 (s, 3H), 2.24 (s, 3H) 1.39 (s, 9H). MS (ESI): calcd for $C_{29}H_{40}ClN_8O_4S$ $[M+H]^+$: 631.26. Found: 631.25.

2-((6-(4-(2-(2-aminoethoxy)ethyl)piperazin-1-yl)-2-methylpyrimidin-4-yl)amino)-*N*-(2-chloro-6-methylphenyl)thiazole-5-carboxamide-TFA salt

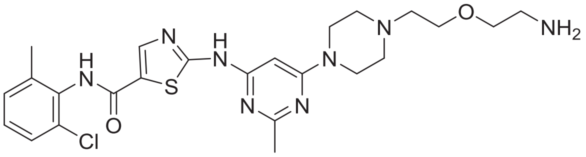

Tert-butyl (2-(2-(4-(6-((5-((2-chloro-6-methylphenyl)carbamoyl)thiazol-2-yl)amino)-2-methylpyrimidin-4-yl)piperazin-1-yl)ethoxy) ethyl)carbamate (75.0 mg, 0.119 mmol) was dissolved in 2.5 mL of DCM and triisopropylsilane (250 μL, 0.031 mmol) and TFA (2.5 mL) was added. The mixture was capped and stirred for 3 hr. Volatiles were removed under reduced pressure giving a yellow oil and 5 mL of diethyl ether was added giving a white precipitate. The ether was decanted and an additional 5 mL portion was added. The suspension was sonicated and the ether was decanted. The resulting residue was dried under high vacuum overnight affording the product (63.1 mg, 100%) as a yellow solid. MS (ESI): calcd for $C_{24}H_{32}ClN_8O_2S$ $[M+H]^+$: 531.21. Found: 531.18.

2-((6-(4-(2-(2-aminoethoxy)ethyl)piperazin-1-yl)-2-methylpyrimidin-4-yl)amino)-*N*-(2-chloro-6-methylphenyl)thiazole-5-carboxamide-NanoBRET 590 (Energy Transfer Probe 6)

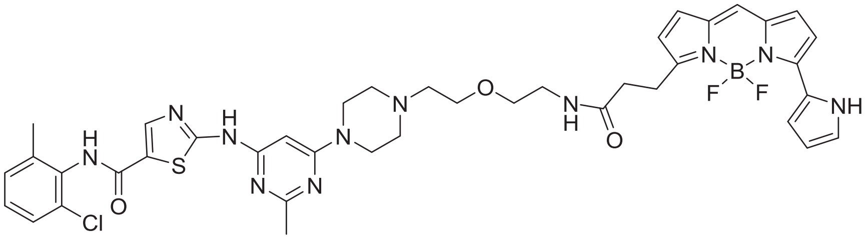

To a solution of 2-((6-(4-(2-(2-aminoethoxy)ethyl)piperazin-1-yl)-2-methylpyrimidin-4-yl)amino)-*N*-(2-chloro-6-methylphenyl)thiazole-5-carboxamide (10.0 mg, 0.024 mmol) in anhydrous DMF (2 mL) was added *N*,*N*-diisopropylethylamine (8.2 μL, 0.047 mmol). The solution was stirred for ten minutes, NanoBRET 590 SE (10.0 mg, 0.024 mmol) was added, the mixture was capped and stirred for 2 hrs, judged complete by the disappearance of the starting amine by HPLC. The reaction was quenched with 6 mL of a solution of acetonitrile, water, TFA (1:1:0.01) and was subjected to RP-HPLC preparative purification using Standard Method 1. Product

containing fractions were analyzed by HPLC for purity, pooled and were concentrated under reduced pressure affording the product (11.6 mg, 73.2%) as a blue film. ^{1}H NMR (400 MHz, d_6 DMSO-*d*) δ 11.46 (s, 1H), 11.41 (s, 1H), 9.87 (s, 1H), 8.21 (s, 1H), 8.01 (t, *J* = 5.7 Hz, 1H), 7.44 (dd, *J* = 2.0 Hz, *J* = 7.5 Hz, 1H), 7.37 (s, 1H), 7.33 (d, *J* = 4.5 Hz, 1H), 7.27 (m, 3H), 7.16 (d, *J* = 4.6 Hz, 1H), 7.01 (d, *J* = 3.9 Hz, 1H), 6.34 (d, *J* = 4.0 Hz, 2H), 6.04 (s, 1H), 3.54 (t, *J* = 6.0 Hz, 2H), 3.50 (s, 4H), 3.43 (t, *J* = 6.0 Hz, 2H), 3.29 (s, 1H), 3.24 (m, 2H), 3.15 (m, 3H), 2.54 (s, 4H), 2.40 (s, 3H), 2.24 (s, 3H). MS (ESI): calcd for $C_{40}H_{44}BClF_2N_{11}O_3S$ $[M+H]^+$: 842.31. Found: 842.24

QUANTIFICATION AND STATISTICAL ANALYSIS

Data from multiple independent experiments (N) are presented as mean values +/- standard error of the mean (SE) and data involving technical replicates are presented as mean +/- standard deviation (SD) as indicated in the figure legends. The number of experimental or technical replicates for each experiment is also described in each individual figure legend. Apparent affinity values were determined using the sigmoidal dose-response (variable slope) equation available in GraphPad Prism (Version 7). Linear regression analyses were determined using Graphpad Prism (Version 7).

Cell Chemical Biology, Volume *25*

Supplemental Information

Quantitative, Wide-Spectrum Kinase Profiling in Live Cells for Assessing the Effect of Cellular ATP on Target Engagement

James D. Vasta, Cesear R. Corona, Jennifer Wilkinson, Chad A. Zimprich, James R. Hartnett, Morgan R. Ingold, Kristopher Zimmerman, Thomas Machleidt, Thomas A. Kirkland, Kristin G. Huwiler, Rachel Friedman Ohana, Michael Slater, Paul Otto, Mei Cong, Carrow I. Wells, Benedict-Tilman Berger, Thomas Hanke, Carina Glas, Ke Ding, David H. Drewry, Kilian V.M. Huber, Timothy M. Willson, Stefan Knapp, Susanne Müller, Poncho L. Meisenheimer, Frank Fan, Keith V. Wood, and Matthew B. Robers

Supplemental Information

Quantitative, Wide-Spectrum Kinase Profiling in Live Cells for Assessing the Effect of Cellular ATP on Target Engagement

Content

Figure S1, Reproducibility of compound IC_{50} measurements for Kinase/NanoLuc fusions, Related to figure 4……………………………………………………………………………………2
Figure S2, Bioluminescence imaging of Nluc fusion proteins and CRISPR experiments, Related to figures 3–6……………………………………………………………………………………4
Figure S3, Analysis of chemical probe 6j, examination of the influence of tracer on test compound affinity for RIPK1, and reproducibility of crizotib profiling, Related to figures 3–6……………………6
Table S2, IC_{50} or K_d values for crizotinib under various assay conditions, Related to figure 5……………8
Figure S4, Reproducibility of kinome profiling with crizotinib and dasatinib, Related to figure 6……………9
Figure S5, Validation of ATP depletion conditions for determination of kinase target engagement, Related to figure 7……………………………………………………………………………………10
Figure S6, Determination of target engagement parameters for kinase/NLuc fusions under ATP depleted conditions, Related to figure 7……………………………………………………………12
Figure S7, Determination of crizotinib or ATP affinity for LCK, EPHB6 and MuSK kinases in permeabilized HEK293 cells using linearized Cheng-Prusoff analysis, Related to figure 7……………14

Figure S1, Related to Figure 4

A

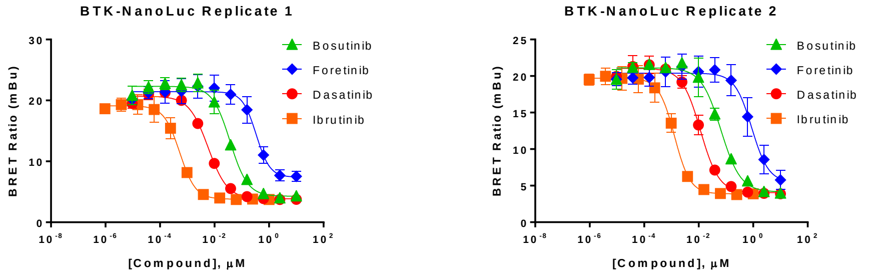

	Bosutinib	Foretinib	Dasatinib	Ibrutinib
LogEC50	0.04124	0.06707	0.02714	0.03892

	Bosutinib	Foretinib	Dasatinib	Ibrutinib
EC50	0.06529	0.884	0.01164	0.001294

B

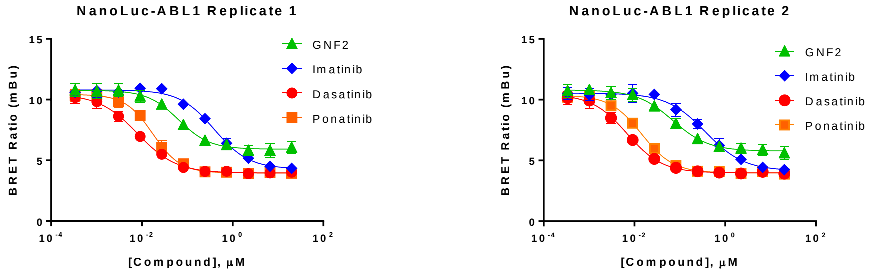

	GNF2	Imatinib	Dasatinib	Ponatinib
EC50	0.0642	0.3947	0.00803	0.01732

	GNF2	Imatinib	Dasatinib	Ponatinib
EC50	0.07024	0.3676	0.006847	0.01443

C

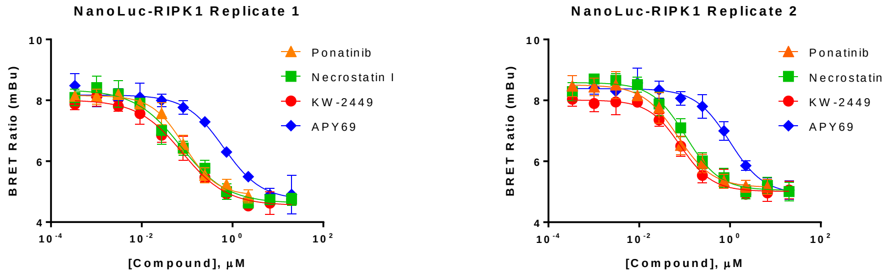

	APY69	KW-2449	Necrostatin I	Ponatinib
EC50	0.6339	0.07844	0.07311	0.08604

	APY69	KW-2449	Necrostatin I	Ponatinib
EC50	0.9828	0.08098	0.1069	0.07028

D

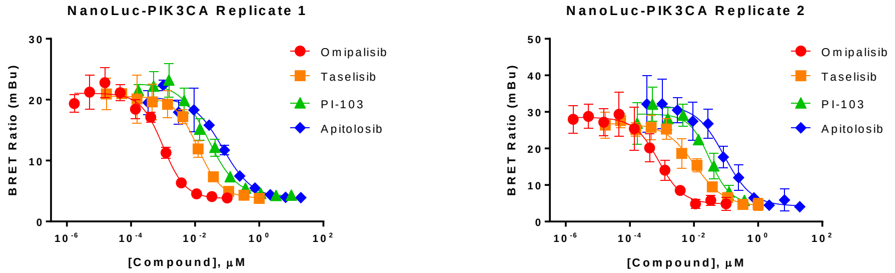

	Omipalisib	Taselisib	PI-103	Apitolosib
EC50	0.0009871	0.01203	0.02588	0.07154

	Omipalisib	Taselisib	PI-103	Apitolosib
EC50	0.0007782	0.01057	0.03054	0.08633

E

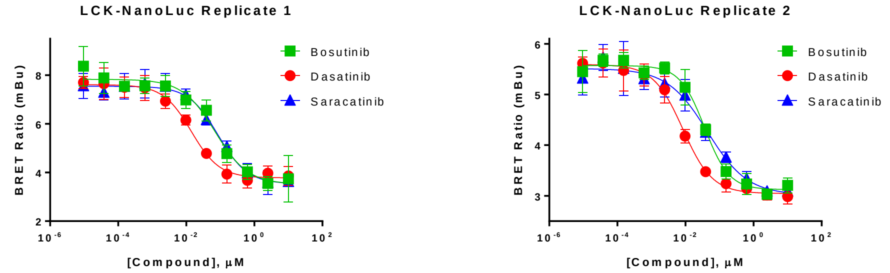

	Bosutinib	Dasatinib	Saracatinib
EC50	0.07079	0.01402	0.08623

	Bosutinib	Dasatinib	Saracatinib
EC50	0.03662	0.008386	0.04445

Figure S1, Reproducibility of compound IC_{50} measurements for Kinase/NanoLuc Fusions, Related to Figure 4. HEK293 cells expressing the kinase/Nluc fusion were seeded into 96-well plates and then treated with a dose-response of compound in the presence of a fixed concentration of energy transfer probe (as described in supplemental table 1). Data in each curve represent the mean (± SD) of 4 technical replicates.

Figure S2, Related to Figures 3–6

A

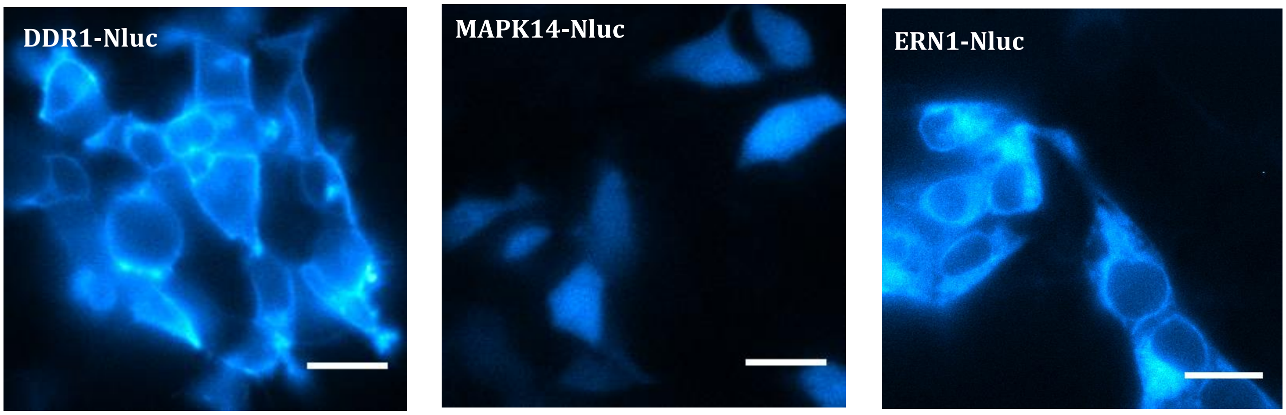

B

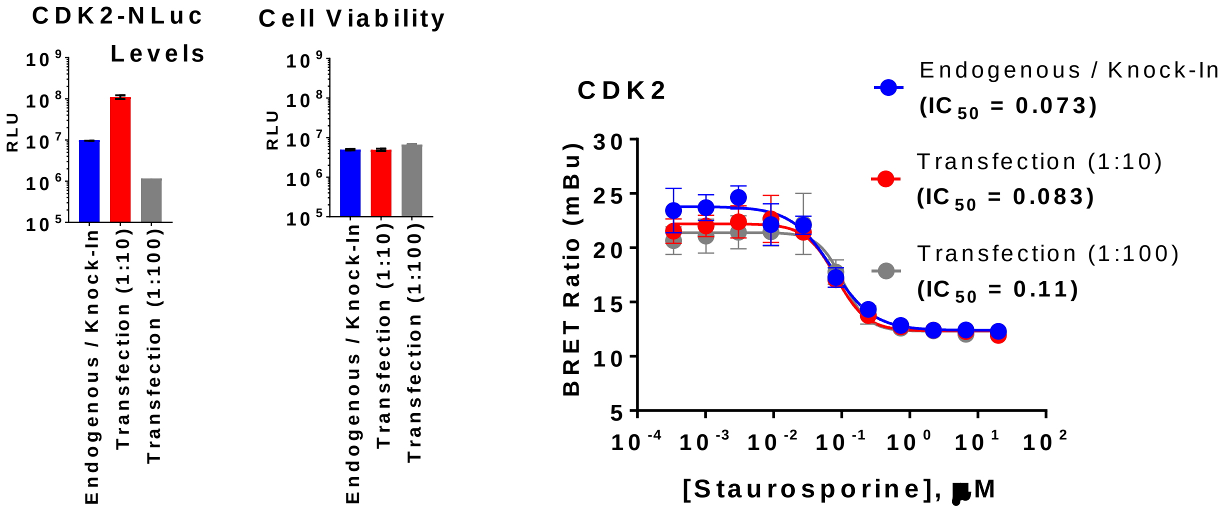

C

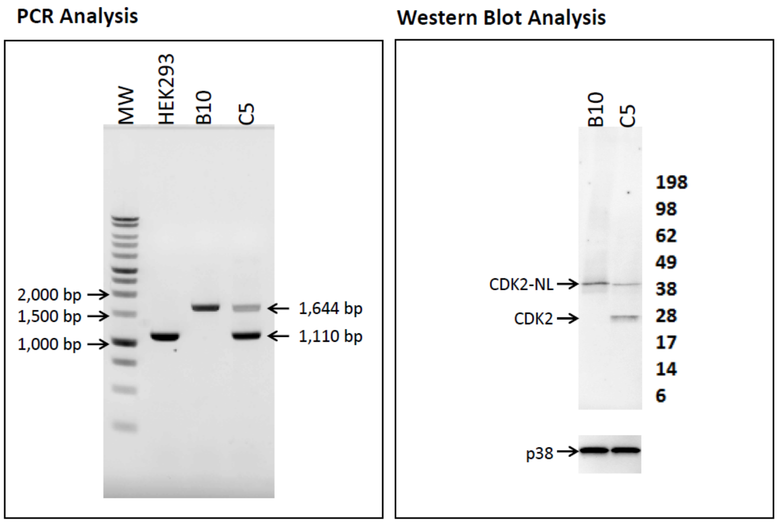

D

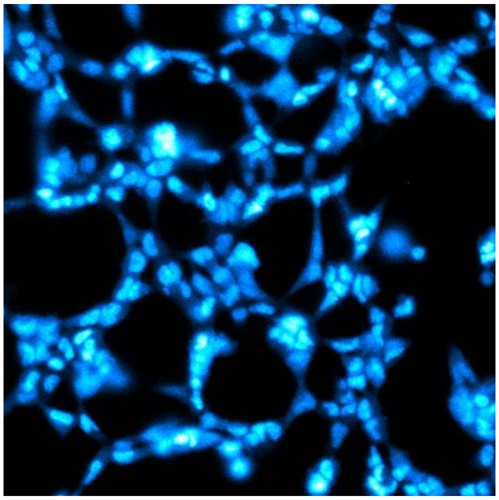

Figure S2. Bioluminescence imaging of Nluc fusion proteins and CRISPR experiments, Related to Figures 3–6. A. Bioluminescence imaging of Nluc fusion proteins. DDR1, MAPK14, and ERN1 (IRE1α) show localization in the plasma membrane, cytoplasm, or endoplasmic reticulum, respectively, thus supporting target engagement analysis in various subcellular compartments. Scale bar = 20 μm. B. Comparison of target engagement at CDK2 in HEK-293 cells, using endogenously expressed (CRISPR-edited) CDK2-NLuc, versus cells transiently expressing CDK2-Nluc proteins. Nluc expression levels with transfected cells were within 10-fold of the expression levels of endogenously-expressed (CRISPR-edited) CDK2, as measured using Nluc luminescence to quantify the relative amounts of CDK2-Nluc (left). To dilute expression levels of CDK2-Nluc, plasmid DNA was diluted into DNA encoding cyclin E from a pF5 plasmid backbone. Cell mass was similar under each condition, as determined using ATP levels via Cell-Titer-Glo (middle). Target engagement potencies with staurosporine were similar under all expression levels (right). Data are the mean (± SD) of 4 technical replicates. C: CRISPR-edited HEK-293 cells expressing CDK2-Nluc are homozygous (clone B10) based on PCR and Western blot analysis. The heterozygous clone (C5) is included for reference. D: In the CRISPR-derived CDK2-Nluc B10 clone, localization of Nluc was predominantly nuclear, consistent with expected localization of CDK2 (Hiromura et al., 2002). Localization was determined using bioluminescence imaging in live cells in the presence of NanoLuc substrate.

Figure S3, Related to Figures 3–6

A

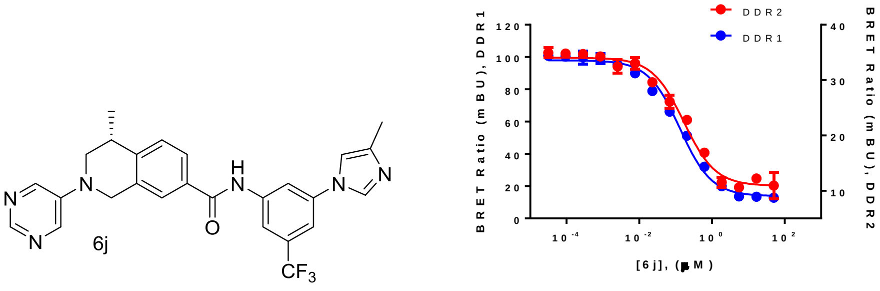

B

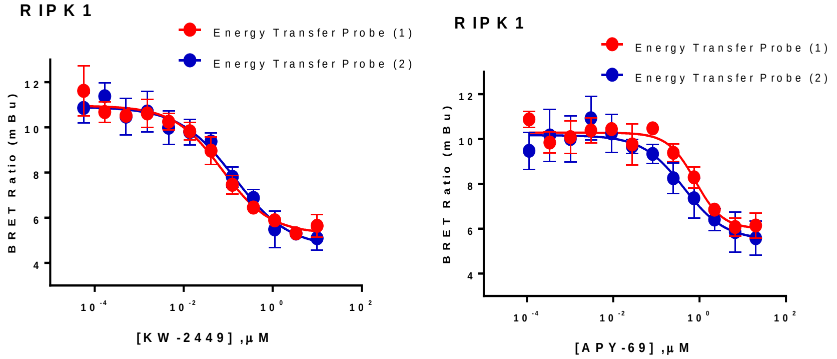

	Probe 1	Probe 2
IC50	0.07318	0.1361

	Probe 1	Probe 2
IC50	0.7914	0.4283

C

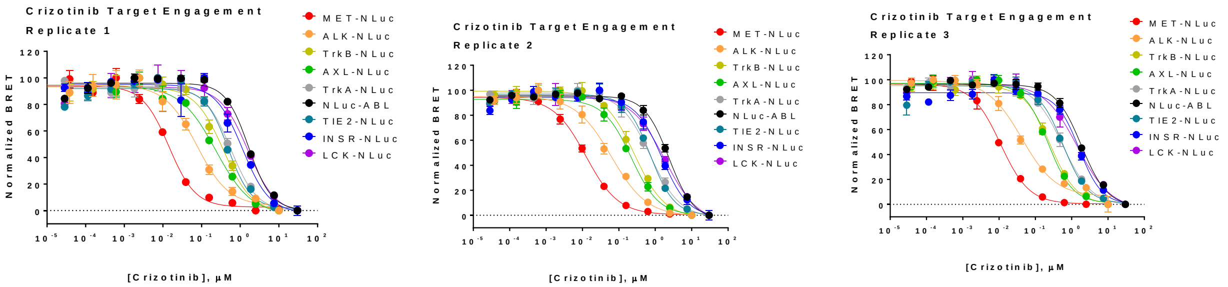

D

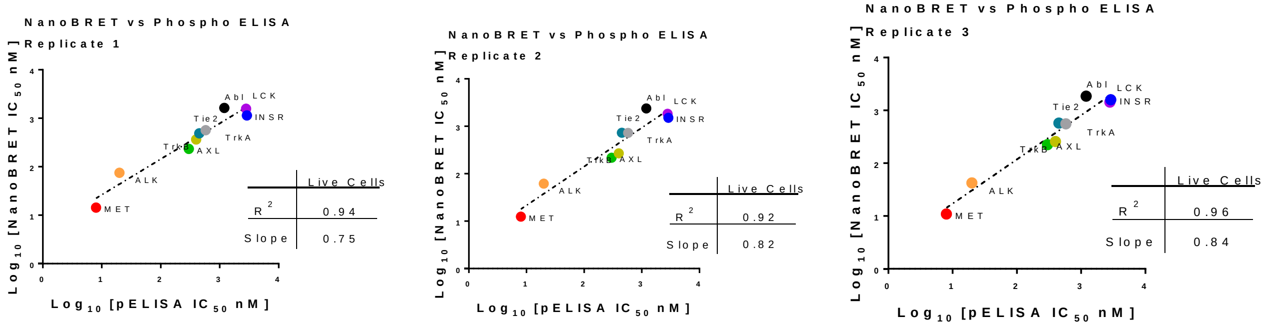

Figure S3. Analysis of chemical probe 6j, examination of the influence of tracer on test compound affinity for RIPK1, and reproducibility of crizotib profiling, Related to Figures 3–6. A. Chemical structure (left) and target engagement potency of 6j for DDR1 vs DDR2 (right) using energy transfer probe 6. Data are mean +/- S.E. for three independent experiments performed in 384-well format. B. Comparative target engagement analysis for RIPK1 using various energy transfer probes. For RIPK1, engagement potency was within a two-fold range for KW-2449 and APY-69 using energy transfer probe 1 or 2. Data are the mean (± SD) of 4 technical replicates. C. Reproducibility of crizotinib target engagement potency for various intracellular kinases. Full data set for crizotinib kinase panel represents three independent experiments, with IC_{50} values for crizotinib at each kinase target tabulated in Table S2. D. IC_{50} values extracted from curve fits are correlated with the corresponding crizotinib IC_{50} as determined by previously by Cui *et al*., 2011, via phospho ELISA after log transformation.

Table S2, IC_{50} or K_d values for Crizotinib Under Various Assay Conditions, Related to Figure 5

Target	Crizotinib K_d Biochemical (nM) [A]	Crizotinib IC_{50} Biochemical (nM) [B]	Live Cell IC_{50} NanoBRET (nM)	Live Cell IC_{50} Phospho Elisa (nM) [B]
**ABL**	110	24	2000 ± 400	1200
**ALK**	3.3	< 1.0	60 ± 20	20
**AXL**	7.8	< 1.0	225 ± 8	300
**INSR**	340	102	1400 ± 200	2900
**TIE2**	270	5.0	600 ± 100	450
**TRKA**	95	< 1.0	600 ± 100	580
**TRKB**	37	2.0	300 ± 60	400
**LCK**	30	< 1.0	1600 ± 200	2800
**MET**	2.1	< 1.0	13 ± 2	8

[A]Data is reproduced from Davis *et al.,* 2011

[B]Data is reproduced from Cui *et al.,* 2011

Figure S4, Related to Figure 6

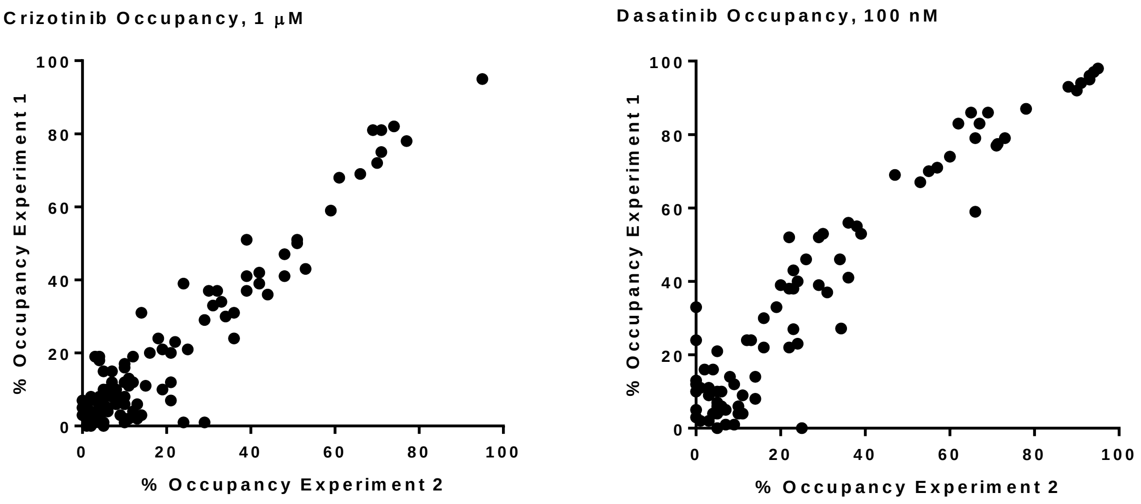

Figure S4, Reproducibility of kinome profiling with crizotinib and dasatinib, Related to Figure 6. HEK293 cells expressing a library of kinase/Nluc fusions were seeded into 96-well plates and then treated with 1 uM crizotinib (left) or 100 nM dasatinib (right) in the presence of energy transfer probe. Data in each graph represent the inter-assay correlation analysis of target occupancy for 2 independent experimental replicates.

Figure S5, Validation of ATP depletion conditions for determination of kinase target engagement, Related to Figure 7

A

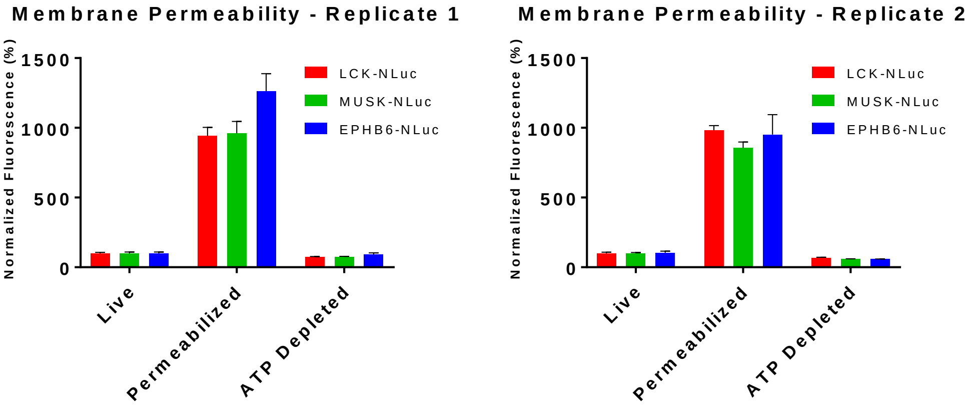

B

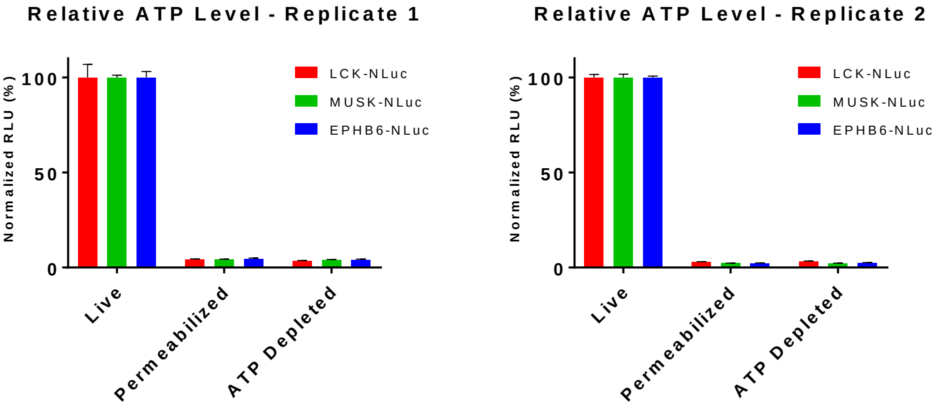

C

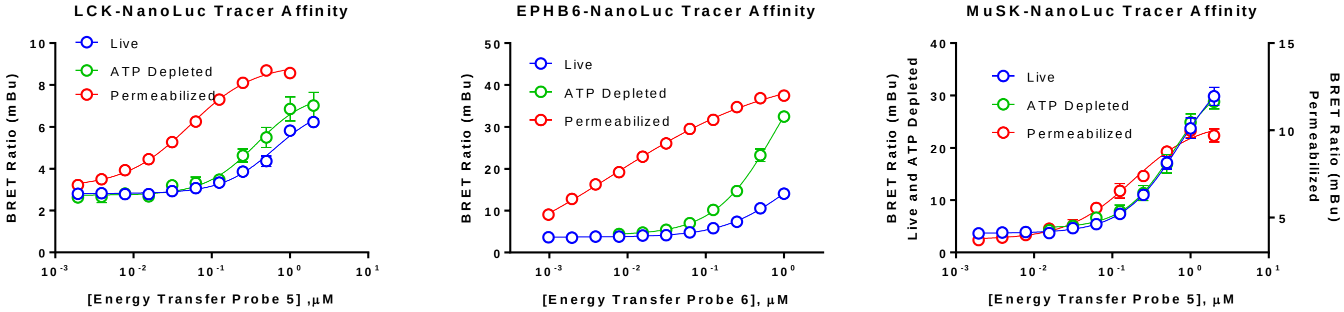

Condition	Live	ATP Depleted	Permeabilized
EC_{50}, μM	> 1	0.37 ± 0.07	0.052 ± 0.003

Condition	Live	ATP Depleted	Permeabilized
EC_{50}, μM	> 1	> 1	0.007 ± 0.003

Condition	Live	ATP Depleted	Permeabilized
EC_{50}, μM	> 1	> 1	0.18 ± 0.02

Figure S5, Validation of ATP depletion conditions for determination of kinase target engagement, Related to Figure 7. A. Validation of live-cell ATP depletion and cell permeabilization conditions**.** HEK293 cells transiently expression the kinase/NLuc fusion protein were subjected to ATP depletion by either inhibition of mitochondrial/glycolytic ATP synthesis (ATP Depleted) or by permeabilization with digitonin (Permeabilized) as described earlier. After treatment for 2h, membrane integrity (Panels A) and relative ATP levels (Panel B) were assessed using CellTox-Green and CellTiter-Glo assays, respectively. Raw fluorescence or luminescence results for all conditions were normalized to that of vehicle treated controls (Live). Individual data sets represent the mean (± SD) of 4 technical replicates. Replicates 1 and 2 represent the results of two independent experiments. Permeabilization with digitonin routinely disrupted membrane integrity (increased relative fluorescence) and reduced total ATP levels (reduced relative RLU) compared to untreated controls. Within 2 hours, ATP depletion by inhibition of mitochondrial/glycolytic ATP synthesis routinely reduced total ATP levels by 90-95% of that for the untreated controls, but without disrupting membrane integrity (no significant increase in relative fluorescence by CellTox-Green). Panel C. Target engagement measurements for crizotinib at kinase/NLuc fusions under ATP depleted conditions. HEK293 cells transiently expressing kinase/NLuc were subjected to vehicle treatment (live), ATP depletion, or permeabilization, after which the target engagement profile of crizotinib was evaluated. To determine a comparable test concentration for subsequent IC_{50} determination, the affinity of energy transfer probe 5 for LCK and MuSK, as well as the affinity of energy transfer probe 6 for EPHB6, was measured under all test conditions. Individual data points represent the mean (± SD) of 4 technical replicates.

Figure S6, Related to Figure 7

A

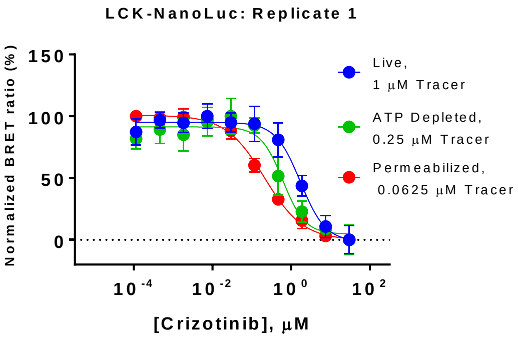

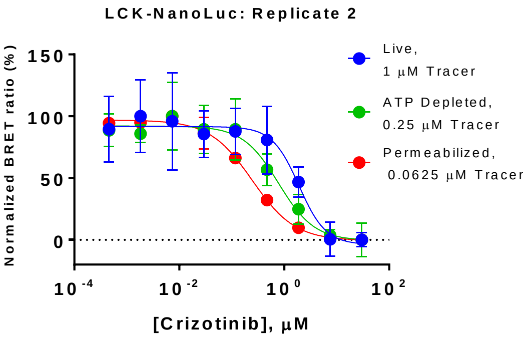

Condition	Live	ATP Depleted	Permeabilized
Crizotinib IC_{50}, nM	1700 ± 300	610 ± 140	210 ± 20

Condition	Live	ATP Depleted	Permeabilized
Crizotinib IC_{50}, nM	1900 ± 600	780 ± 270	250 ± 20

B

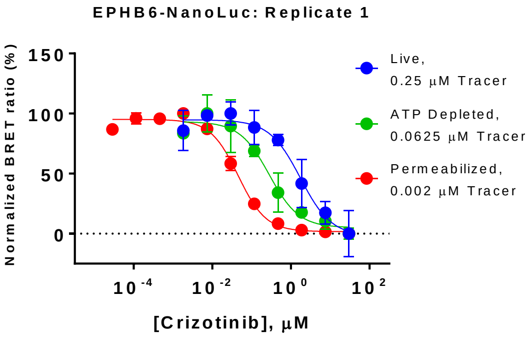

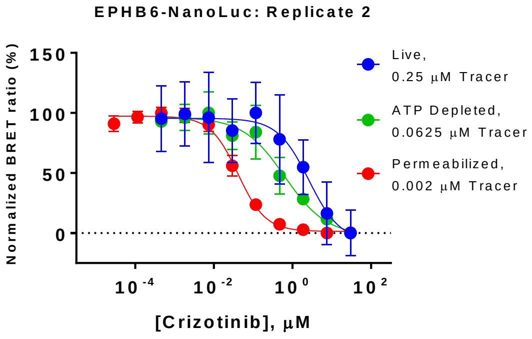

Condition	Live	ATP Depleted	Permeabilized
Crizotinib IC_{50}, nM	1700 ± 600	280 ± 90	45 ± 4

Condition	Live	ATP Depleted	Permeabilized
Crizotinib IC_{50}, nM	3000 ± 2000	630 ± 260	41 ± 4

C

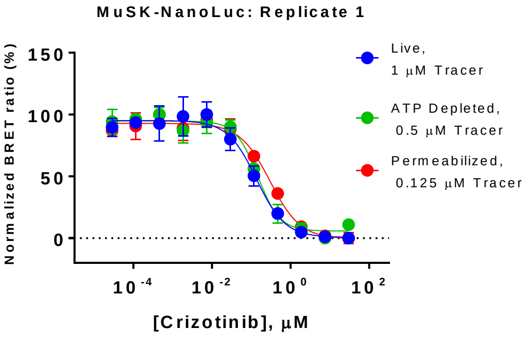

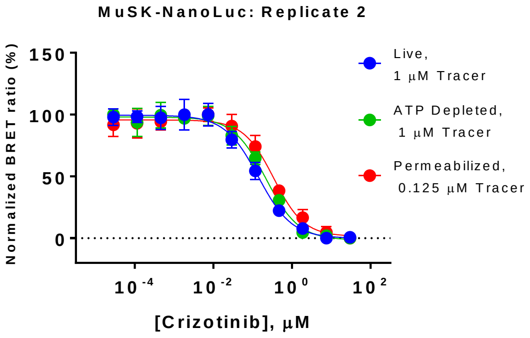

Condition	Live	ATP Depleted	Permeabilized
Crizotinib IC_{50}, nM	140 ± 20	150 ± 20	300 ± 40

Condition	Live	ATP Depleted	Permeabilized
Crizotinib IC_{50}, nM	140 ± 20	220 ± 30	300 ± 40

D

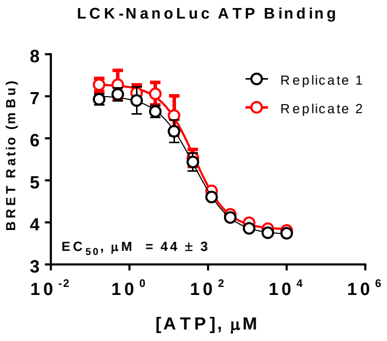

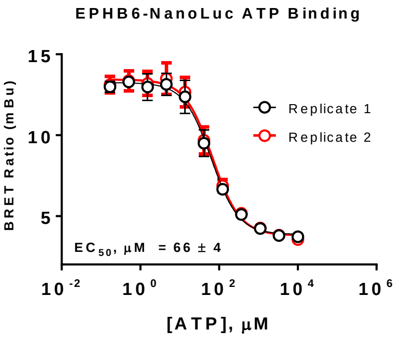

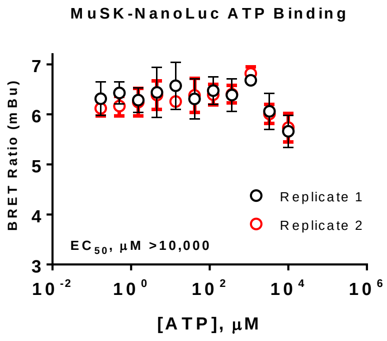

Figure S6. Determination of target engagement parameters for kinase/NLuc fusions under ATP depleted conditions, Related to Figure 7. Panels A–C. Crizotinib IC_{50} measurements for LCK (A) and EPHB6 (B) under ATP depleted and permeabilized conditions showed significant left shifted potency compared to the live cell controls, but that of MuSK (C) was consistent between 3 conditions. Individual data points represent the mean (± SD) of 4 technical replicates, with replicates 1 and 2 depicting the results of two independent measurements. D. LCK (Panel and EPHB6 showed significant ATP binding in permeabilized cells, but MuSK did not. Individual data points represent the mean (± SD) of 4 technical replicates. Replicates 1 and 2 represent the results of two independent experiments.

Figure S7, Related to Figure 7

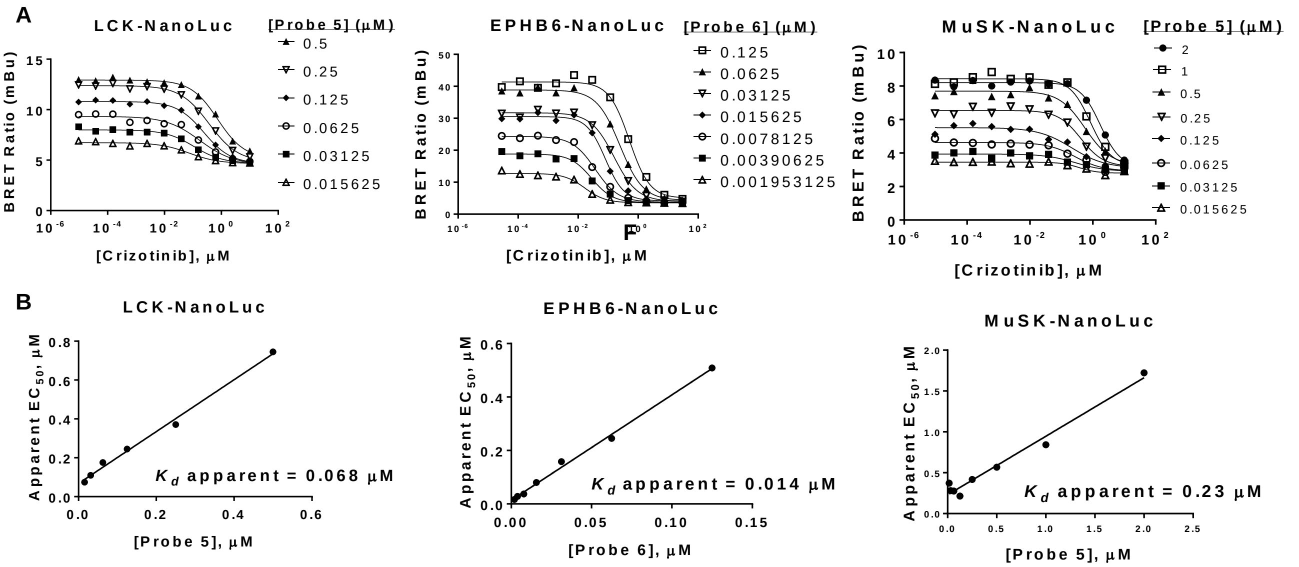

Target	LCK	EPHB6	MuSK
Crizotinib K_d, nM<br>Permeabilized HEK293 Cells	70 ± 10	14 ± 6	230 ± 40
Crizotinib K_i, nM<br>Biochemical (Davis, *et al*. 2011)	30	6	230

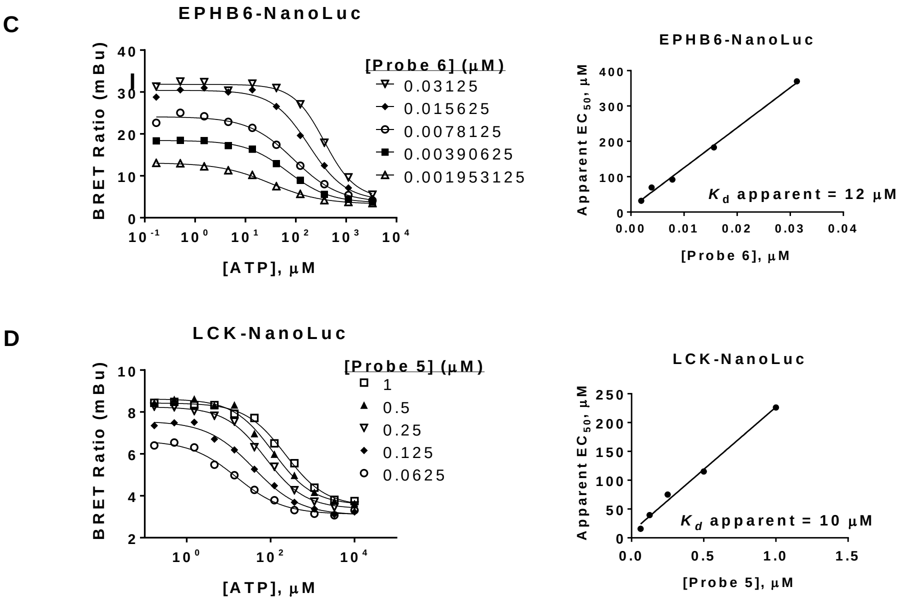

LCK Replicate	1	2	3	4	Mean
ATP K_d (μM)	10.49	19.62	15.37	15.69	15
Error	5.51	5.06	7.54	5.96	3

Figure S7, Determination of crizotinib or ATP affinity for LCK, EPHB6 and MuSK kinases in permeabilized HEK293 cells using linearized Cheng-Prusoff analysis, Related to Figure 7. A. Determination of crizotinib affinity. HEK293 cells expressing kinase/NanoLuc fusions were seeded into 96-well plates, permeabilized with digitonin, and then treated with a dose-response of crizotinib at increasing concentrations of energy transfer probe. (Panel B) Apparent IC_{50} values for crizotinib were replotted versus the energy transfer probe concentration, and the K_d value for crizotinib was determined from the y-intercept by linear regression. Individual data points are the mean (± SD) of 4 technical replicates. K_d values (± SE) for crizotinib are provided in the accompanying table, and are in agreement with biochemical K_i values reported previously (Davis *et al*., 2011). (Panel C) Determination of ATP affinity for EPHB6-NanoLuc. HEK293 cells expressing EPHB6-NanoLuc were seeded into 96-well plates, permeabilized with digitonin, and then treated with a dose-response of rATP at increasing concentrations of energy transfer probe 6. Apparent IC_{50} values for rATP were replotted versus the energy transfer probe concentration, and the K_d value for rATP was determined from the y-intercept by linear regression. Though a pseudokinase, EPHB6 has been shown previously to bind rATP using a thermal shift assay (Murphy *et al*., 2014). (Panel D) Determination of ATP affinity for LCK-NanoLuc. HEK293 cells expressing LCK-NanoLuc were seeded into 96-well plates, permeabilized with digitonin, and then treated with a dose-response of rATP at increasing concentrations of energy transfer probe 5 (1–0.0625 μM). Apparent IC_{50} values for rATP were replotted versus the energy transfer probe concentration, and the K_d value for rATP was determined from the y-intercept by linear regression. Replicates 1–4 represent 4 independent experiments, and the individual K_d values for rATP are provided in the accompanying table. The mean K_d of ATP for LCK-NanoLuc is in agreement with the biochemical ATP K_M reported previously (Knight and Shokat, 2005).
